# Supplementary material for: Coordinating power sector climate transitions under policy uncertainty
Source: Nat Commun. 2025 Apr 22;16:3786. doi: 10.1038/s41467-025-59126-1 (PMC12015370; doi:10.1038/s41467-025-59126-1)
Supplement: Supplementary file 1 — Supplementary Information [file 41467_2025_59126_MOESM1_ESM.pdf]

Coordinating Power Sector Climate Transitions Under Policy Uncertainty

Supplementary Information

Nomenclature

Sets

$\mathcal{C}$ Eligible resources for CES

$\mathcal{E}$ Economic dispatch units

$\mathcal{G}$ All resources

$\mathcal{H}$ Reservoir hydro units

$\mathcal{L}$ Existing transmission paths

$\mathcal{M}$ Segments in demand curtailment strategy

$\mathcal{N}$ Non-dispatchable resources

$\mathcal{R}$ Run-of-river hydro units

$\mathcal{S}$ Storage units

$\mathcal{T}$, $\mathcal{T}_b$, $\mathcal{T}_i$ All, beginning, and interior hours of sub-periods, respectively

$\mathcal{U}$ Unit commitment resources

$\mathcal{W}$ Eligible resources for RPS

$\mathcal{Z}$ Zones

Indices

$g \in \mathcal{G}$ A resource

$l \in \mathcal{L}$ A transmission path

$m \in \mathcal{M}$ A segment in demand curtailment strategy

$t \in \mathcal{T}$ An hour in the modeling horizon

$z \in \mathcal{Z}$ A zone

Parameters

α_z Zonal RPS targets in percent of generation

β_z, β Zonal and region-wide reserve margin in percent of peak demand

$\breve{\pi}_g$ Startup cost of resource g (\$/MW)

χ_z, χ Zonal and region-wide contingencies for operating reserve

δ In-state RPS and CES requirements (fraction)

Δ_g, Δ_g^e Power and energy capacities of existing resource g

Δ_l Capacity of existing transmission path l (MW)

η_g^+, η_g^- Charge and discharge efficiencies for resource g

Γ_g Initial and final state of charge for resource g in percent of installed energy capacity

γ_{zt} Net international import for zone z at time period t (MW)

κ_g^+, κ_g^- Maximum ramp-up/down rates for resource g in percent of installed capacity

Λ_m Maximum allowed non-served demand for segment m in percent of total demand

μ_g^-, μ_g^+ Minimum and maximum discharge duration for resource g

ω_{gt} Capacity factor for resource g at time period t in percent of capacity

Ω_g Potential of resource g (MW)

$\omega_g^{'}$, $\omega_g^{''}$ Intercept and coefficient to estimate maximum power output for resource g

$\overline{d}_z$, $\overline{d}$ Zonal and region-wide peak demands (MW)

π_g, π_g^e Investment cost per MW and per MWh for resource g (\$/MW-year, \$/MWh-year)

$\pi_g^{''}$ Variable cost for resource g (\$/MWh)

$\pi_g^{'}$, $\pi_g^{'e}$ Fixed O&M cost per MW and per MWh for resource g (\$/MW-year, \$/MWh-year)

π_l Investment cost for existing path l (\$/MW-year)

$\pi_l^{'}$ Fixed O&M cost for path l (\$/MW-year)

ρ_{gt} Time-dependent minimum output for resource g in percent of capacity

ρ_g Minimum stable output for resource g in percent of capacity

$\rho_g^{'}$, $\rho_g^{''}$ Intercept and coefficient to estimate minimum power output for resource g

σ_z Hurdle rate cost for exporting power from zone z (\$/MWh)

τ_g^+, τ_g^- Minimum up/down time for resource g

$\tilde{\pi}_m$ Cost of non-served energy in segment m (\$/MW)

ξ_z, ξ Zonal and region-wide CES targets in percent of generation

a_g Minimum reservoir level for resource g in percent of energy capacity

d_{zt} Electricity demand in zone z at time period t (MW)

$e_z^{\leftarrow\prime}$, $e_z^{\rightarrow\prime}$ Increased fixed import/export to meet planning reserve for zone z (MW)

$e_z^{\leftarrow}$, $e_z^{\rightarrow}$ Fixed import/export to meet planning reserve for zone z (MW)

f_{gt} Inflow amount for resource g at time period t in percent of capacity

h Number of hours in a sub-period

j_z, j Zonal and region-wide demand contribution to operating reserve requirement (%)

k_z, k Zonal and region-wide generation contribution to operating reserve requirement (%)

w_t Sample weight for time period t

x_g Number of units for resource g in a cluster

y_g Unit size of resource g (MW)

Decision variables

$C_{gt} \in \mathbb{R}_+$ Power used for charging resource g at time period t

$D_g \in \mathbb{R}_+$, $D_g^{\mathbb{Z}} \in \mathbb{Z}_+$ Retired capacity and number of retired units of resource g

$E_{gt} \in \mathbb{R}_+$ State of charge of resource g at time period t

$F_{lt} \in \mathbb{R}$ Flow in path l at time period t (MW)

$G_{gt} \in \mathbb{R}_+$ Generation output of resource g (MW) at time period t

$H_{lt} \in \mathbb{R}_+$ Total hurdle rate cost in path l at time period t

$I_g \in \mathbb{R}_+$, $I_g^{\mathbb{Z}} \in \mathbb{Z}_+$ Installed capacity and number of installed units of resource g

$I_g^e, D_g^e, J_g^e \in \mathbb{R}_+$ Energy capacity of resource g, tied to I_g, D_g, and J_g

$I_l \in \mathbb{R}_+$ Added capacity in existing path l

$J_g \in \mathbb{R}_+$, $J_g^{\mathbb{Z}} \in \mathbb{Z}_+$ Capacity and number of units of resource g, not subject to retirement

$M_{zmt} \in \mathbb{R}_+$ Non-served demand in zone z for curtailment segment m at time period t

$O_g \in \mathbb{R}_+$, $O_g^e \in \mathbb{R}_+$ Final power and energy capacities of resource g

$O_l \in \mathbb{R}_+$: Final capacity of transmission path l (MW)

$R_{gt}^+ \in \mathbb{R}_+$, $R_{gt}^- \in \mathbb{R}_+$ Up/down reserve contributions from resource g at time period t

$R_{gt}^{\leftarrow+} \in \mathbb{R}_+$, $R_{gt}^{\leftarrow-} \in \mathbb{R}_+$ Up/down reserve contributions from resource g while charging

$R^{\rightarrow +}_{gt} \in \mathbb{R}_+$, $R^{\rightarrow -}_{gt} \in \mathbb{R}_+$ Up/down reserve contributions from resource g while discharging

$U_{gt} \in \mathbb{Z}_+$ Commitment status of resource g at time period t

$V^{\uparrow}_{gt} \in \mathbb{Z}_+$, $V^{\downarrow}_{gt} \in \mathbb{Z}_+$ Startup and shutdown events for resource g at time period t

1 Clarification for notations

1.1 Sets

Each resource group (unit commitment, economic dispatch, storage, reservoir hydro, and run-of-river) combines new build, retirable existing, and non-retirable existing units. We define the set of unit commitment resources with $\mathcal{U} \triangleq \{\mathcal{U}_n,\mathcal{U}_o,\mathcal{U}_c\}$ where $\mathcal{U}_n$, $\mathcal{U}_o$, and $\mathcal{U}_c$ refer to sets of new build, retirable existing, and non-retirable existing units, respectively. We have the same definitions for $\mathcal{E}$, $\mathcal{S}$, $\mathcal{H}$, $\mathcal{R}$, and $\mathcal{G}$. Set $\mathcal{G}$ is defined with $\mathcal{G} \triangleq \mathcal{U} \cup \mathcal{E} \cup \mathcal{S} \cup \mathcal{H}$. We treat run-of-river units as typical non-dispatchable resources ($\mathcal{R} \subset \mathcal{E} \subset \mathcal{G}$). $\mathcal{C}$ includes all CES-eligible resources, excluding RPS-eligible resources. Hence, $\mathcal{W} \cup \mathcal{C}$ defines all CES-eligible resources.

Notations with subscript z refer to elements in zone z. Particular to lines, $\mathcal{L}_z$ refers to transmission lines connecting zone z. We denote dispatchable resources not subject to unit commitment constraints with $\mathcal{G} \setminus \{\mathcal{U} \cup \mathcal{N}\}$. Similarly, we denote non-dispatchable renewable resources with $\mathcal{W} \cap \mathcal{N}$.

Table 1 lists technologies included in resource sets. Sets $\mathcal{H}$ and $\mathcal{R}$ include reservoir hydro and run-of-river units, respectively.

1.2 Parameters

Zonal peak demand ($\overline{d}_z$) is the maximum demand over all hours for zone z. Region-wide peak demand ($\overline{d}$) is the maximum total coincident demand over all zones over all hours; i.e., $\overline{d} = \max_t \sum_{z \in \mathcal{Z}} d_{zt}$. In planning reserve constraints, we multiply installed capacities of resources with the capacity factor attained at the hour at which region-wide peak demand

Set	Included technologies
$\mathcal{C}$	CCS coal, CCS gas, nuclear
$\mathcal{E}$	Solar, solar thermal with energy storage, wind, run-of-river hydro, geothermal
$\mathcal{N}$	Solar, wind, run-of-river hydro
$\mathcal{S}$	Battery, hydroelectric pumped storage, solar thermal with energy storage
$\mathcal{U}$	Biomass[1], coal, gas, nuclear, other peaker[2]
$\mathcal{W}$	Biomass, solar, solar thermal with energy storage, wind, reservoir hydro, run-of-river hydro, geothermal

[1] Biomass is a grouped technology. It includes wood/wood waste biomass, landfill gas, municipal solid waste, and other waste biomass. These are names used in EIA database [1].

[2] 'Other peaker' is a grouped technology. It includes natural gas internal combustion engines, petroleum liquids, petroleum coke, 'other gases', 'other natural gas', and 'all other'. 'All other', 'other gases', and 'other natural gas' are names used in EIA database.

Supplemental Table 1: Resources and technologies

occurs. In other words, we find $\omega_{g,\arg\max_t \sum_z d_{zt}}$.

2 Constraints

2.1 Fixed decision variables

For existing resources not subject to retirement, we fix J at the capacity or at the number of units.

$$J_g = \Delta_g \qquad \forall g \in \{\mathcal{E}_c, \mathcal{S}_c, \mathcal{H}_c\} \tag{1}$$

$$J_g^e = \Delta_g^e \qquad \forall g \in \{\mathcal{S}_c, \mathcal{H}_c\} \tag{2}$$

$$J_g^{\mathbb{Z}} = x_g \qquad \forall g \in \mathcal{U}_c \tag{3}$$

2.2 Constraints on auxiliary variables

The final capacities of resources (O_g) are determined by expansion and retirement.

$$O_g = I_g \qquad \forall g \in \{\mathcal{E}_n, \mathcal{S}_n, \mathcal{H}_n\} \tag{4}$$

$$O_g = y_g I_g^{\mathbb{Z}} \qquad \forall g \in \mathcal{U}_n \tag{5}$$

$$O_g = \Delta_g - D_g \qquad \forall g \in \{\mathcal{E}_o, \mathcal{S}_o, \mathcal{H}_o\} \tag{6}$$

$$O_g = y_g(x_g - D_g^{\mathbb{Z}}) \qquad \forall g \in \mathcal{U}_o \tag{7}$$

$$O_g = J_g \qquad \forall g \in \{\mathcal{E}_c, \mathcal{S}_c, \mathcal{H}_c\} \tag{8}$$

$$O_g = y_g J_g^{\mathbb{Z}} \qquad \forall g \in \mathcal{U}_c \tag{9}$$

$$O_g^e = I_g^e \qquad \forall g \in \{\mathcal{S}_n, \mathcal{H}_n\} \tag{10}$$

$$O_g^e = \Delta_g^e - D_g^e \qquad \forall g \in \{\mathcal{S}_o, \mathcal{H}_o\} \tag{11}$$

$$O_g^e = J_g^e \qquad \forall g \in \{\mathcal{S}_c, \mathcal{H}_c\} \tag{12}$$

2.3 Constraints on retirements

Retired capacities should be less than existing capacities.

$$D_g \leq \Delta_g \qquad \forall g \in \{\mathcal{E}_o, \mathcal{S}_o, \mathcal{H}_o\} \tag{13}$$

$$D_g^{\mathbb{Z}} \leq x_g \qquad \forall g \in \mathcal{U}_o \tag{14}$$

$$D_g^e \leq \Delta_g^e \qquad \forall g \in \{\mathcal{S}_o, \mathcal{H}_o\} \tag{15}$$

For a storage unit or reservoir hydro unit, if either power or energy capacity is retired, the other should also be retired in proportion to discharge duration.

$$D_g^e = D_g(\Delta_g^e/\Delta_g) \qquad \forall g \in \{\mathcal{S}_o, \mathcal{H}_o\} \tag{16}$$

2.4 Constraints on installations

Potential refers to the maximum installation amount of geographically constrained resources. These limits are based on land availability and the availability of solar, wind, water, and geothermal heat.

$$I_g \leq \Omega_g \qquad \forall g \in \{\mathcal{E}_n, \mathcal{H}_n\} \tag{17}$$

The energy capacity (or duration) of candidate storage units is decided by the model.

$$I^e_g \geq \mu^-_g I_g \qquad \forall g \in \{\mathcal{S}_n, \mathcal{H}_n\} \tag{18}$$

$$I^e_g \leq \mu^+_g I_g \qquad \forall g \in \{\mathcal{S}_n, \mathcal{H}_n\} \tag{19}$$

2.5 Expansion on transmission paths

The final capacity of each path is the sum of existing capacity and additional capacity being decided by the model. We do not put any upper bound for the capacity expansion.

$$O_l = \Delta_l + I_l \qquad \forall l \in \mathcal{L} \tag{20}$$

2.6 Constraints on generator output

Variable renewable energy units cannot produce more power than weather conditions allow. Unit commitment resources produce power driven by their commitment status.

$$G_{gt} \leq \omega_{gt} O_g \qquad \forall g \in \{\mathcal{E}, \mathcal{S}, \mathcal{H}\}, \forall t \in \mathcal{T} \tag{21}$$

$$G_{gt} \leq O_g \qquad \forall g \in \mathcal{H}, \forall t \in \mathcal{T} \tag{22}$$

$$G_{gt} \leq \omega_{gt} y_g U_{gt} \qquad \forall g \in \mathcal{U}, \forall t \in \mathcal{T} \tag{23}$$

Note that $\omega_{gt} = 1$ for all unit commitment resources. For reservoir hydro, we determine intercept and linear coefficients for maximum power output in terms of inflows (Equation 104). We need Constraint (22) because ω_{gt} sometimes results in numbers larger than 1.

$$G_{gt} \geq \rho_g O_g \qquad \forall g \in \{\mathcal{E}, \mathcal{S}\}, \forall t \in \mathcal{T} \tag{24}$$

$$G_{gt} \leq \rho_{gt} O_g \qquad \forall g \in \mathcal{H}, \forall t \in \mathcal{T} \tag{25}$$

$$G_{gt} \geq \rho_g y_g U_{gt} \qquad \forall g \in \mathcal{U}, \forall t \in \mathcal{T} \tag{26}$$

Power output from resources is also bounded below. $\rho_g = 0$ for all economic dispatch and storage units. For reservoir hydro units, we calculate ρ_{gt} as a linear function of inflow amount (Equation 103).

2.7 Ramping constraints

Unit commitment resources have additional constraints on operations. Here, we show constraints relating to ramp-up limitations. κ_g^+ equals 1 for all resources not subject to unit commitment operations.

$$G_{gt} - G_{g,t-1} \leq \kappa_g^+ O_g \qquad \forall g \in \{\mathcal{E}, \mathcal{S}, \mathcal{H}\}, \forall t \in \mathcal{T}_i \tag{27}$$

$$G_{gt} - G_{g,t+h-1} \leq \kappa_g^+ O_g \qquad \forall g \in \{\mathcal{E}, \mathcal{S}, \mathcal{H}\}, \forall t \in \mathcal{T}_b \tag{28}$$

$$G_{gt} - G_{g,t-1} \leq \rho_g y_g (U_{gt} - U_{g,t-1}) + \kappa_g^+ y_g U_{gt} \qquad \forall g \in \mathcal{U}, \forall t \in \mathcal{T}_i \tag{29}$$

$$G_{gt} - G_{g,t+h-1} \leq \rho_g y_g (U_{gt} - U_{g,t+h-1}) + \kappa_g^+ y_g U_{gt} \qquad \forall g \in \mathcal{U}, \forall t \in \mathcal{T}_b \tag{30}$$

Similarly, ramp-down constraints are

$$G_{g,t-1} - G_{gt} \leq \kappa_g^- O_g \qquad \forall g \in \{\mathcal{E}, \mathcal{S}, \mathcal{H}\}, \forall t \in \mathcal{T}_i \tag{31}$$

$$G_{g,t+h-1} - G_{gt} \leq \kappa_g^- O_g \qquad \forall g \in \{\mathcal{E}, \mathcal{S}, \mathcal{H}\}, \forall t \in \mathcal{T}_b \tag{32}$$

$$G_{g,t-1} - G_{gt} \leq \rho_g y_g (U_{g,t-1} - U_{gt}) + \kappa_g^- y_g U_{gt} \qquad \forall g \in \mathcal{U}, \forall t \in \mathcal{T}_i \tag{33}$$

$$G_{g,t+h-1} - G_{gt} \leq \rho_g y_g (U_{g,t+h-1} - U_{gt}) + \kappa_g^- y_g U_{gt} \qquad \forall g \in \mathcal{U}, \forall t \in \mathcal{T}_b \tag{34}$$

2.8 Constraints on non-served energy amount

Even though it is an impractical and undesired outcome of a power system, demand may not always be satisfied. We have multiple segments of non-served demand and demand response management, each of which corresponds to a maximum allowed unmet amount and cost.

$$M_{zmt} \leq \Lambda_m d_{zt} \qquad \forall z \in \mathcal{Z}, \forall m \in \mathcal{M}, \forall t \in \mathcal{T} \tag{35}$$

$$\sum_{m \in \mathcal{M}} M_{zmt} \leq d_{zt} \qquad \forall z \in \mathcal{Z}, \forall t \in \mathcal{T} \tag{36}$$

2.9 Storage unit operations

The energy level of a storage unit is determined by the energy level from the previous period and the dispatched and charged energy amount during the current period. We consider efficiencies of discharging and charging operations.

$$E_{gt} = E_{g,t-1} + \eta_g^+ C_{gt} - G_{gt}/\eta_g^- \qquad \forall g \in \mathcal{S}, \forall t \in \mathcal{T}_i \tag{37}$$

$$E_{gt} = E_{g,t+h-1} + \eta_g^+ C_{gt} - G_{gt}/\eta_g^- \qquad \forall g \in \mathcal{S}, \forall t \in \mathcal{T}_b \tag{38}$$

The energy level of storage units is capacitated.

$$E_{gt} \leq O_g^e \qquad \forall g \in \{\mathcal{S}, \mathcal{H}\}, \forall t \in \mathcal{T} \tag{39}$$

We fix the initial and the final energy levels of storage units and make them equivalent.

$$E_{gt} = \Gamma_g O_g^e \qquad \forall g \in \{\mathcal{S}, \mathcal{H}\}, \forall t \in \{1, |\mathcal{T}|\} \tag{40}$$

Constraints (39) and (40) apply to reservoir hydro units because they behave similar to storage. Energy balance constraints are slightly modified for reservoir hydro by replacing charged amount with the inflow parameter.

$$E_{gt} = E_{g,t-1} + \eta_g^+ f_{gt} O_g - G_{gt}/\eta_g^- \qquad \forall g \in \mathcal{H}, \forall t \in \mathcal{T}_i \tag{41}$$

$$E_{gt} = E_{g,t+h-1} + \eta_g^+ f_{gt} O_g - G_{gt}/\eta_g^- \qquad \forall g \in \mathcal{H}, \forall t \in \mathcal{T}_b \tag{42}$$

Reservoir levels have minimum levels.

$$E_{gt} \geq a_g O_g^e \qquad \forall g \in \mathcal{H}, \forall t \in \mathcal{T} \tag{43}$$

We model existing solar thermal with energy storage units as storage and they are charged from the grid. They are not charged with available solar power due to the lack of inflow time series. We treat new build solar thermal with energy storage units as solar projects with long dispatch durations, which makes them dispatchable units. We do not model them as storage.

2.10 Unit commitment state equations

We utilize a three-variable formulation to model thermal units' operations, further clustered into integers [2]. The following constraints determine the commitment status of clustered thermal units:

$$U_{gt} \leq I_g^{\mathbb{Z}} \qquad \forall g \in \mathcal{U}_n, \forall t \in \mathcal{T} \tag{44}$$

$$U_{gt} \leq x_g - D_g^{\mathbb{Z}} \qquad \forall g \in \mathcal{U}_o, \forall t \in \mathcal{T} \tag{45}$$

$$U_{gt} \leq J_g \qquad \forall g \in \mathcal{U}_c, \forall t \in \mathcal{T} \tag{46}$$

We have the same constraints for start-up and shut-down operations.

$$V_{gt}^{\uparrow} \leq I_g^{\mathbb{Z}} \qquad \forall g \in \mathcal{U}_n, \forall t \in \mathcal{T} \tag{47}$$

$$V_{gt}^{\uparrow} \leq x_g - D_g^{\mathbb{Z}} \qquad \forall g \in \mathcal{U}_o, \forall t \in \mathcal{T} \tag{48}$$

$$V_{gt}^{\uparrow} \leq J_g \qquad \forall g \in \mathcal{U}_c, \forall t \in \mathcal{T} \tag{49}$$

$$V_{gt}^{\downarrow} \leq I_g^{\mathbb{Z}} \qquad \forall g \in \mathcal{U}_n, \forall t \in \mathcal{T} \tag{50}$$

$$V_{gt}^{\downarrow} \leq x_g - D_g^{\mathbb{Z}} \qquad \forall g \in \mathcal{U}_o, \forall t \in \mathcal{T} \tag{51}$$

$$V_{gt}^{\downarrow} \leq J_g \qquad \forall g \in \mathcal{U}_c, \forall t \in \mathcal{T} \tag{52}$$

Lastly, the relationship between commitment status and start-up/shut-down status should be defined.

$$U_{gt} - U_{g,t-1} = V_{gt}^{\uparrow} - V_{gt}^{\downarrow} \qquad \forall g \in \mathcal{U}, \forall t \in \mathcal{T} \setminus \{1\} \tag{53}$$

2.11 Time limits for thermal units' up and down status

Once thermal units start operating, they must run for at least a specified number of hours. Similarly, they must stay non-operating for at least a specified number of hours once they are shut down.

$$U_{gt} \geq \sum_{t'=t-\tau_g^+}^{t} V_{gt'}^{\uparrow} \qquad \forall g \in \mathcal{U}, \forall t \in \mathcal{T} \tag{54}$$

$$I_g^{\mathbb{Z}} - U_{gt} \geq \sum_{t'=t-\tau_g^-}^{t} V_{gt'}^{\downarrow} \qquad \forall g \in \mathcal{U}_n, \forall t \in \mathcal{T} \tag{55}$$

$$x_g - D_g^{\mathbb{Z}} - U_{gt} \geq \sum_{t'=t-\tau_g^-}^{t} V_{gt'}^{\downarrow} \qquad \forall g \in \mathcal{U}_o, \forall t \in \mathcal{T} \tag{56}$$

$$J_g^{\mathbb{Z}} - U_{gt} \geq \sum_{t'=t-\tau_g^-}^{t} V_{gt'}^{\downarrow} \qquad \forall g \in \mathcal{U}_c, \forall t \in \mathcal{T} \tag{57}$$

These constraints do not work if time indexing start with 1. Users need to take precautions such as redefining $\forall t \in \mathcal{T}$ as $\forall t \in \{\max_g \tau_g, \max_g \tau_g + 1, ..., \mathcal{T}\}$.

2.12 Constraints on power flow amounts

Flow on transmission lines is bounded by line capacities.

$$F_{lt} \leq O_l \qquad \forall l \in \mathcal{L}, \forall t \in \mathcal{T} \tag{58}$$

$$F_{lt} \geq -O_l \qquad \forall l \in \mathcal{L}, \forall t \in \mathcal{T} \tag{59}$$

2.13 Demand-balance constraint

Demand and supply are balanced for each modeling zone. The sum of dispatched power and net international import (equal to 0 for non-border zones) should equal the sum of demand, power used to charge storage units, and power exported to other regions. We consider non-served demand in this equation. Note that power transfer between zones ($\phi_{zl} F_{lt}$) may take positive or negative signs. If positive (negative), it refers to export (import).

$$\sum_{g \in \mathcal{G}_z} G_{gt} + \gamma_{zt} + \sum_{m \in \mathcal{M}} M_{zmt} = \sum_{g \in \mathcal{S}_z} C_{gt} + d_{zt} + \sum_{l \in \mathcal{L}} \phi_{zl} F_{lt} \qquad \forall z \in \mathcal{Z}, \forall t \in \mathcal{T} \tag{60}$$

Here, ϕ_{zl} is a parameter indicating whether zone z is directly connected with path l (1: power flows from zone z, 0: zone z not connected, -1: power flows to zone z).

2.14 Reserve contributions from resources

For unit commitment resources, we have

$$R_{gt}^{+} \leq y_g U_{gt} \qquad \forall g \in \mathcal{U}, \forall t \in \mathcal{T} \tag{61}$$

$$R_{gt}^{+} \leq y_g U_{gt} - G_{gt} \qquad \forall g \in \mathcal{U}, \forall t \in \mathcal{T} \tag{62}$$

$$R_{gt}^{+} \leq \kappa_g^{+} y_g U_{gt} \qquad \forall g \in \mathcal{U}, \forall t \in \mathcal{T} \tag{63}$$

$$R_{gt}^{-} \leq y_g U_{gt} \qquad \forall g \in \mathcal{U}, \forall t \in \mathcal{T} \tag{64}$$

$$R_{gt}^{-} \leq G_{gt} - \rho_g y_g U_{gt} \qquad \forall g \in \mathcal{U}, \forall t \in \mathcal{T} \tag{65}$$

$$R_{gt}^{-} \leq \kappa_g^{-} y_g U_{gt} \qquad \forall g \in \mathcal{U}, \forall t \in \mathcal{T} \tag{66}$$

A resource can provide up reserve total size of committed resources (Constraint 61) at the most. The provided reserve is limited by the remaining capacity of committed resources if they dispatch power (Constraint 62). The provided reserve cannot exceed the ramping limit (Constraint 63). These constraints have the same interpretation for down reserve, except that dispatched power can be reduced until the minimum power limit (Constraint 65).

For dispatchable non-unit commitment resources, we have

$$R_{gt}^{+} \leq \omega_{gt} O_g \qquad \forall g \in \{\mathcal{G} \setminus \{\mathcal{U} \cup \mathcal{N}\}\} \setminus \mathcal{H}, \forall t \in \mathcal{T} \tag{67}$$

$$R_{gt}^{+} \leq \omega_{gt} O_g - G_{gt} \qquad \forall g \in \{\mathcal{G} \setminus \{\mathcal{U} \cup \mathcal{N}\}\} \setminus \{\mathcal{H} \cup \mathcal{S}\}, \forall t \in \mathcal{T} \tag{68}$$

$$R_{gt}^{-} \leq \omega_{gt} O_g \qquad \forall g \in \{\mathcal{G} \setminus \{\mathcal{U} \cup \mathcal{N}\}\} \setminus \mathcal{H}, \forall t \in \mathcal{T} \tag{69}$$

$$R_{gt}^{-} \leq G_{gt} \qquad \forall g \in \{\mathcal{G} \setminus \{\mathcal{U} \cup \mathcal{N}\}\} \setminus \{\mathcal{H} \cup \mathcal{S}\}, \forall t \in \mathcal{T} \tag{70}$$

The provided reserve cannot exceed the weather-dependent limit (Constraints 67 and 69). The reserve amount cannot be larger than the remaining capacity when resources dispatch power (Constraint 68). Dispatch amount can be reduced completely to provide down reserve (Constraint 70).

For reservoir hydro units, we have

$$R_{gt}^{+} \leq \kappa_g^{+} O_g \qquad \forall g \in \mathcal{H}, \forall t \in \mathcal{T} \qquad (71)$$

$$R_{gt}^{+} \leq \omega_{gt} O_g - G_{gt} \qquad \forall g \in \mathcal{H}, \forall t \in \mathcal{T} \qquad (72)$$

$$R_{gt}^{+} \leq E_{gt} - a_g O_g^{e} - G_{gt} \qquad \forall g \in \mathcal{H}, \forall t \in \mathcal{T} \qquad (73)$$

$$R_{gt}^{-} \leq \kappa_g^{-} O_g \qquad \forall g \in \mathcal{H}, \forall t \in \mathcal{T} \qquad (74)$$

$$R_{gt}^{-} \leq G_{gt} - \rho_{gt} O_g \qquad \forall g \in \mathcal{H}, \forall t \in \mathcal{T} \qquad (75)$$

Reserves should be less than ramping limit (Constraints 71 and 74). Up reserves should be less than the remaining capacity when resources dispatch power (Constraint 72). Dispatched power can be reduced until the minimum power boundary to provide down reserve (Constraint 75). Constraint (73) is an active constraint if the state of charge is low. Reservoir hydro units cannot dispatch power (or provide up reserves) more than its remaining energy capacity in an hour.

For storage resources, the up reserve amount is the sum of reserves provided during charging and discharging. The same applies to down reserve as well.

$$R_{gt}^{+} = R_{gt}^{\leftarrow +} + R_{gt}^{\rightarrow +} \qquad \forall g \in \mathcal{S}, \forall t \in \mathcal{T} \qquad (76)$$

$$R_{gt}^{-} = R_{gt}^{\leftarrow -} + R_{gt}^{\rightarrow -} \qquad \forall g \in \mathcal{S}, \forall t \in \mathcal{T} \qquad (77)$$

Provided reserves by storage are driven by charging and discharging decisions [3]. Providing down reserve while charging means absorbing more power from the network. Provided down reserve can be at the most either power capacity or remaining energy capacity of storage units, considering charging efficiency. Provided up reserve while charging means allocation of charging effort to dispatch power, and thus reserve amount can be charging amount at the most.

$$C_{gt} + R_{gt}^{\leftarrow -} \leq O_g / \eta_g^{+} \qquad \forall g \in \mathcal{S}, \forall t \in \mathcal{T} \qquad (78)$$

$$C_{gt} + R_{gt}^{\leftarrow -} \leq (O_g^{e} - E_{gt}) / \eta_g^{+} \qquad \forall g \in \mathcal{S}, \forall t \in \mathcal{T} \qquad (79)$$

$$C_{gt} - R_{gt}^{\leftarrow +} \geq 0 \qquad \forall g \in \mathcal{S}, \forall t \in \mathcal{T} \qquad (80)$$

Provided up reserve while discharging is limited by either power capacity or state of charge, considering discharging efficiency [3]. Provided down reserve while discharging is bounded by discharging amount.

$$G_{gt} + R_{gt}^{\rightarrow +} \leq \eta_g^- O_g \qquad \forall g \in \mathcal{S}, \forall t \in \mathcal{T} \tag{81}$$

$$G_{gt} + R_{gt}^{\rightarrow +} \leq \eta_g^- E_{g,t-1} \qquad \forall g \in \mathcal{S}, \forall t \in \mathcal{T} \tag{82}$$

$$G_{gt} - R_{gt}^{\rightarrow -} \geq 0 \qquad \forall g \in \mathcal{S}, \forall t \in \mathcal{T} \tag{83}$$

Storage investments are continuous, indicating that storage capacity represents the aggregated capacity of multiple individual storage units. In this case, simultaneous discharging and charging at any period are possible [3].

$$(G_{gt} + R_{gt}^{\rightarrow +})/\eta_g^- + \eta_g^+(C_{gt} + R_{gt}^{\leftarrow -}) \leq O_g \qquad \forall g \in \mathcal{S}, \forall t \in \mathcal{T} \tag{84}$$

In addition to the formulation by [3], provided total up reserve is limited by power capacity considered with discharge efficiency.

$$R_{gt}^{+} \leq \eta_g^- O_g \qquad \forall g \in \mathcal{S}, \forall t \in \mathcal{T} \tag{85}$$

Non-dispatchable resources can provide only down reserve. We have

$$R_{gt}^{-} \leq G_{gt} \qquad \forall g \in \mathcal{N}, \forall t \in \mathcal{T} \tag{86}$$

Also, run-of-river hydro units can only provide down reserve. We have

$$R_{gt}^{-} \leq \kappa_g^- O_g \qquad \forall g \in \mathcal{R}, \forall t \in \mathcal{T} \tag{87}$$

$$R_{gt}^{-} \leq G_{gt} - \rho_g O_g \qquad \forall g \in \mathcal{R}, \forall t \in \mathcal{T} \tag{88}$$

2.15 Operating reserve requirements

2.15.1 BAU scenario

Up reserve requirement in each zone for each time period is a certain percentage of demand plus a certain percentage of total dispatch from non-dispatchable renewable resources plus a

contingency. We calculate the contingency of a zone (χ_z) as the maximum of the following three parameters: (i) the largest unit size of existing thermal resources in this zone, (ii) the largest capacity of power lines connecting this zone, and (iii) the largest unit size of candidate thermal resources in this zone.

$$\sum_{g \in \mathcal{U}_z \cup \{\mathcal{G}_z \setminus \{\mathcal{U}_z \cup \mathcal{N}_z\}\}} R^+_{gt} \geq j_z d_{zt} + k_z \sum_{g \in \{\mathcal{W}_z \cap \mathcal{N}_z\} \setminus \mathcal{R}_z} G_{gt} + \chi_z \qquad \forall z \in \mathcal{Z}, \forall t \in \mathcal{T} \tag{89}$$

For down reserve, we have

$$\sum_{g \in \mathcal{G}_z} R^-_{gt} \geq j_z d_{zt} + k_z \sum_{g \in \{\mathcal{W}_z \cap \mathcal{N}_z\} \setminus \mathcal{R}_z} G_{gt} \qquad \forall z \in \mathcal{Z}, \forall t \in \mathcal{T} \tag{90}$$

Differences between up and down reserve formulations are the resources contributing to reserve and contingency. All resources in zone z provide a down reserve, and contingency is not required.

2.15.2 Expanded EIM and regional market scenarios

Operating reserve requirements are met at the regional level. We compute the contingency of region as the maximum of the following three parameters: (i) the largest unit size of existing thermal resources, (ii) the largest capacity of power lines, and (iii) the largest unit size of candidate thermal resources.

$$\sum_{g \in \mathcal{U} \cup \{\mathcal{G} \setminus \{\mathcal{U} \cup \mathcal{N}\}\}} R^+_{gt} \geq j \sum_{z \in \mathcal{Z}} d_{zt} + k \sum_{g \in \{\mathcal{W} \cap \mathcal{N}\} \setminus \mathcal{R}} G_{gt} + \chi \qquad \forall t \in \mathcal{T} \tag{91}$$

For down reserve, we have

$$\sum_{g \in \mathcal{G}} R^-_{gt} \geq j \sum_{z \in \mathcal{Z}} d_{zt} + k \sum_{g \in \{\mathcal{W} \cap \mathcal{N}\} \setminus \mathcal{R}} G_{gt} \qquad \forall t \in \mathcal{T} \tag{92}$$

2.16 Planning reserve

2.16.1 BAU scenario

Installed capacity in each zone should exceed peak demand with a margin, considered with the capacity factor. We take into account fixed import and export as well.

$$\sum_{g \in \mathcal{G}_z} \omega_{g,\arg\max_t \sum_z d_{zt}} O_g + e_z^{\leftarrow} - e_z^{\rightarrow} \geq (1+\beta_z)\overline{d}_z \qquad \forall z \in \mathcal{Z} \tag{93}$$

2.16.2 Expanded EIM scenario

Fixed import and export increases.

$$\sum_{g \in \mathcal{G}_z} \omega_{g,\arg\max_t \sum_z d_{zt}} O_g + e_z^{\leftarrow'} - e_z^{\rightarrow'} \geq (1+\beta_z)\overline{d}_z \qquad \forall z \in \mathcal{Z} \tag{94}$$

2.16.3 Regional market scenario

Planning reserve is met region-wide.

$$\sum_{g \in \mathcal{G}} \omega_{g,\arg\max_t \sum_z d_{zt}} O_g \geq (1+\beta)\overline{d} \tag{95}$$

2.17 Hurdle rates

2.17.1 BAU scenario

Hurdle rates are a cost penalty associated with flows between specified zones. Costs may be asymmetric, i.e., flow from zone A to B may not have the same penalty as flow from B to A. This requires us to define a new decision variable, H_{lt}.

$$H_{lt} \geq \sigma_z \phi_{zl} F_{lt} \qquad \forall z \in \mathcal{Z}, \forall l \in \mathcal{L}, \forall t \in \mathcal{T} \tag{96}$$

2.18 RPS and CES constraints

2.18.1 State policy scenario

We enforce the model to meet state-level RPS and CES targets.

$$\sum_{g \in \mathcal{W}_z} \sum_{t \in \mathcal{T}} w_t G_{gt} \geq \delta \alpha_z \sum_{g \in \mathcal{G}_z \setminus \mathcal{S}_z} \sum_{t \in \mathcal{T}} w_t G_{gt} \qquad \forall z \in \mathcal{Z} \tag{97}$$

$$\sum_{g \in \{\mathcal{W}_z \cup \mathcal{C}_z\}} \sum_{t \in \mathcal{T}} w_t G_{gt} \geq \delta \xi_z \sum_{g \in \mathcal{G}_z \setminus \mathcal{S}_z} \sum_{t \in \mathcal{T}} w_t G_{gt} \qquad \forall z \in \mathcal{Z} \tag{98}$$

Recall that δ represents in-state RPS and CES requirements for all zones. Out-of-state imports also contribute to meeting RPS and CES targets. Hence, across the entire region, we have

$$\sum_{z\in\mathcal{Z}}\sum_{g\in\mathcal{W}_z}\sum_{t\in\mathcal{T}} w_t G_{gt} \geq \sum_{z\in\mathcal{Z}}\sum_{g\in\mathcal{G}_z\setminus\mathcal{S}_z}\sum_{t\in\mathcal{T}} \alpha_z w_t G_{gt} \tag{99}$$

$$\sum_{z\in\mathcal{Z}}\sum_{g\in\{\mathcal{W}_z\cup\mathcal{C}_z\}}\sum_{t\in\mathcal{T}} w_t G_{gt} \geq \sum_{z\in\mathcal{Z}}\sum_{g\in\mathcal{G}_z\setminus\mathcal{S}_z}\sum_{t\in\mathcal{T}} \xi_z w_t G_{gt} \tag{100}$$

2.18.2 Regional 100% CES scenario

If a regional 100% CES target should be met, then

$$\sum_{g\in\{\mathcal{W}\cup\mathcal{C}\}}\sum_{t\in\mathcal{T}} w_t G_{gt} \geq \xi \sum_{g\in\mathcal{G}\setminus\mathcal{S}}\sum_{t\in\mathcal{T}} w_t G_{gt} \tag{101}$$

where $\xi = 1$, but it may be equal to other fractions if less stringent regional CES targets are modeled.

3 Objective function

$$\sum_{g\in\mathcal{G}_n} \pi_g I_g + \sum_{g\in\mathcal{G}} \pi_g^{'} O_g + \tag{102a}$$

$$\sum_{g\in\{\mathcal{S}_n\cup\mathcal{H}_n\}} \pi_g^e I_g^e + \sum_{g\in\{\mathcal{S}\cup\mathcal{H}\}} \pi_g^{'e} O_g^e + \tag{102b}$$

$$\sum_{l\in\mathcal{L}} \pi_l I_l + \sum_{l\in\mathcal{L}} \pi_l^{'} O_l + \tag{102c}$$

$$\sum_{g\in\mathcal{G}}\sum_{t\in\mathcal{T}} w_t \pi_g^{''} G_{gt} + \tag{102d}$$

$$\sum_{z\in\mathcal{Z}}\sum_{m\in\mathcal{M}}\sum_{t\in\mathcal{T}} w_t \tilde{\pi}_m M_{zmt} + \tag{102e}$$

$$\sum_{g\in\mathcal{U}}\sum_{t\in\mathcal{T}} \breve{\pi}_g y_g V_{gt}^{\uparrow} + \tag{102f}$$

$$\sum_{l\in\mathcal{L}}\sum_{t\in\mathcal{T}} H_{lt} \tag{102g}$$

Investment and fixed O&M costs per MW for resources (Expression 102a), investment and fixed O&M costs per MWh for storage units (Expression 102b), and investment and

fixed O&M costs for existing transmission paths (Expression 102c) are incurred. The total variable costs from all generators is accounted (Expression 102d). The total variable cost of a generator (\$/MWh) is variable O&M cost (\$/MWh) plus total fuel cost (\$/MWh). Total fuel cost is fuel cost (\$/MMBtu) multiplied by heat rate (MMBtu/MWh). The cost of non-served demand and demand response is added (Expression 102e). The total start cost from all thermal plants is incurred (Expression 102f). The total start cost of a thermal plant (\$/MW) is the start cost (\$/MW) plus the total fuel cost resulting from the start (\$/MW). Total fuel cost is fuel cost (\$/MMBtu) multiplied by fuel amount burned during the start operation (MMBtu/MW). In the BAU scenario, hurdle rate cost is added (Expression 102g).

4 Computational setup

We develop our models in Julia [4], use JuMP for mathematical formulations [5], and benefit from Gurobi as optimization engine [6]. The optimality gap is 0.01% and the time limit is 25.5 hours (all runs terminate within 2-3 hours). We enforce Gurobi to use the barrier method to find a relaxed solution in the initial node of the branch-and-bound tree. We scale the objective function by dividing each element by 10,000 to improve the performance of the optimization engine. We also remove small numbers from the capacity factor data set for the same purpose (i.e., replace all numbers less than 0.01 with 0).

We run our models on the San Diego Supercomputing Center, a high-performance and data-intensive computing and cyber-infrastructure system at the University of California San Diego [7]. We use 16 nodes in computing and set wall time as 26 hours.

5 Input Data

We run PowerGenome [8] to create 12 zones in WECC and to select 6 representative weeks (including peak week) from a year. We create our zones based on regions considered in the Integrated Planning Model (IPM) of Environmental Protection Agency (EPA) [9]. North California covers IPM regions of balancing authorities CALN and BANC; South California

covers SCE and LADW; San Diego covers SDG&E and IID; and Nevada covers NNV and SNV. The rest of the modeling zones each represent one IPM region.

From PowerGenome, we utilize existing generators and storage units with their operation characteristics (e.g., ramp up/down rates), new build generators and storage units with their investment costs, the capacity factors for variable renewable resources, the estimated load in 2050 for all zones, transmission network (e.g., capacity, distance), and fuel prices. In addition, we collect the following datasets. We also list significant data which we input in the PowerGenome settings file.

5.1 Charge and discharge efficiency for storage units

Battery, hydroelectric pumped storage, and solar thermal with energy storage have charge and discharge efficiencies of 0.92, 0.87, and 0.92, respectively [10, supplemental data].

5.2 Minimum/maximum discharge duration for new build storage units

We assume for new build batteries that minimum and maximum discharge duration are 1 and 10 hours, respectively [10, supplemental data]. These numbers are 1 and 12 for new build hydroelectric pumped storage [11; 10; 12]. New build reservoir hydro units have a minimum 1-hour and maximum 10-hour discharge duration.

New build solar thermal with energy storage has maximum discharge duration of 10 hours [13]. We assume that these resources are dispatchable. Moreover, we fix their capacity factor at 40% for all time periods [14]. In future, we may model the operations of these resources in more elaborated ways [15].

5.3 Costs for new build storage units

We assume fixed O&M costs for new build batteries are 1.5% of investment costs [11]. Hence, they are \$2,430/MW and \$3,360/MWh. Equivalent annualized costs are \$224/MW and \$310/MWh, with a service life of 20 years and weighted average capital cost (WACC)

of 6.77% [11].

Capital cost for new build hydroelectric pumped storage is entirely in terms of capacity, given as $2,511,000/MW. The equivalent annualized cost is $187,487/MW, with a service life of 50 years and WACC of 7.24% [11]. We use annualized fixed O&M cost per MW as $25,000 [11]. Lastly, we assume that fixed O&M costs per MWh equal zero.

5.4 Energy capacity of existing storage and reservoir hydro units

To our knowledge, maximum discharge duration of existing storage units does not exist in public data sources. Instead of reporting duration, we find the MWh capacities.

64 batteries located in WECC are listed with their MW and MWh capacities [1]. A list of large-sized energy storage plants worldwide is presented [16], 11 of which are in the WECC service area. We combine these two lists without double-counting. We calculate the maximum discharge duration of each resource by dividing MWh by MW and taking an average duration over all resources. This operation is based on our assumption that all batteries provided by PowerGenome have a single duration equal to what we calculate as average above.

Global hydroelectric pumped storage resources are listed with their basic information [17], only two of which are in the WECC area. Using a similar calculation method adopted for batteries, we assign a single maximum discharge duration for all hydroelectric pumped units in the PowerGenome data set. For solar thermal with energy storage, three units are in WECC area [1], which coincide with the list in [16]. We follow a similar calculation conducted before and assign a single maximum discharge duration to thermal units in the PowerGenome data set. We use discharge duration to compute the MWh capacities of existing storage units by multiplying MW capacities with duration. Battery, hydroelectric pumped storage, and solar thermal with energy storage have a duration of 3.231, 7.963, and 6 hours, respectively.

PowerGenome labels all hydro units as run-of-river if the capacity does not exceed 30

MW, which is a user-adjusted value. To our knowledge, energy capacities of existing reservoir hydro resources do not exist in public data sources. We benefit from an approach presented in [10] to assign reasonable MWh capacities. It is assumed that resources have MWh capacities twice the average hourly inflow over 2009 in California, Arizona, Nevada, Colorado, and New Mexico [10]. Resources in Oregon, Washington, Idaho, Montana, Utah, and Wyoming have MWh capacities four times the average hourly inflow over 2009. However, this approach, in our case, usually brings about MWh capacities smaller than MW capacities. We adjust this assumption so that resources in California, Arizona, Nevada, Colorado, and New Mexico have MWh capacities eight times larger than average hourly inflow; and resources in Oregon, Washington, Idaho, Montana, Utah, and Wyoming have MWh capacities of 16 times larger than average hourly inflow (Table 2).

**Zones**	**MW**	**MWh**	**Zones**	**MW**	**MWh**
Arizona	2,765	5,990	Colorado	483	973
Idaho	1,411	10,420	Montana	2,498	17,553
Nevada	1,039	5,602	North California	7,345	16,683
Pacific Northwest	29,779	224,130	South California	1,424	1,756
Utah	162	833	Wyoming	234	1,254

Supplemental Table 2: Power (MW) and energy (MWh) capacities of reservoir hydro units

5.5 Minimum/maximum power output for reservoir hydro and new build units

We use $\rho_g^{'}$ and $\rho_g^{''}$ to calculate time-dependent minimum output for reservoir hydro units as a linear function of inflow (Equation 103). Similarly, we use $\omega_g^{'}$ and $\omega_g^{''}$ to find maximum dispatchable power as a linear function of inflow (Equation 104). Note that inflow is a percentage of total installed capacity.

RESOLVE publishes a part of input data as a zipped folder in [18]. It includes time series of daily energy budget (inflow) and minimum/maximum power outputs of reservoir hydro resources over 37 days. It shows these times series at the zonal level for CAISO, LDWP, BANC, IID, and aggregated regions of Northwest and Southwest. We assign these to

respective zones in our model, which is more disaggregated outside of California. RESOLVE's aggregated zones are different than ours. CAISO overlaps with South California; BANC with North California; IID with San Diego; Northwest with Colorado, Idaho, Montana, Pacific Northwest, Utah, and Wyoming; and Southwest with Arizona, New Mexico, and Nevada. We perform linear regression analysis on these data sets, considering daily energy budget is the independent variable, and minimum/maximum power outputs are response variables. (Table 3).

$$\rho_{gt} = \rho^{'}_{g} + \rho^{''}_{g} f_{gt} \tag{103}$$

$$\omega_{gt} = \omega^{'}_{g} + \omega^{''}_{g} f_{gt} \tag{104}$$

**Zones**	**Minimum Power** **Intercept** ($\rho^{'}_{g}$)	**Minimum Power** **Coefficient** ($\rho^{''}_{g}$)	**Maximum Power** **Intercept** ($\omega^{'}_{g}$)	**Maximum Power** **Coefficient** ($\omega^{''}_{g}$)
Arizona	-0.0112	0.5227	0.3774	1.0063
Colorado	-0.1615	0.8602	0.328	0.7374
Idaho	-0.1615	0.8602	0.328	0.7374
Montana	-0.1615	0.8602	0.328	0.7374
New Mexico	-0.0112	0.5227	0.3774	1.0063
Nevada	-0.0112	0.5227	0.3774	1.0063
North California	0.008	0.4416	0.2365	0.9413
Pacific Northwest	-0.1615	0.8602	0.328	0.7374
San Diego	0.0073	0.5146	0.2405	1.0034
South California	-0.0868	0.919	0.1919	0.9435
Utah	-0.1615	0.8602	0.328	0.7374
Wyoming	-0.1615	0.8602	0.328	0.7374

Supplemental Table 3: Linear functions to compute minimum/maximum power outputs for reservoir hydro units

Example usage of Table 3 is as follows: Assume that inflow is 30% of installed capacity for reservoir hydro resources in Arizona on January 10, 2050. Also, assume that the total capacity of these units is 1,000 MW. Then, minimum and maximum dispatchable power amounts are 14.56% ($-0.0112 + 0.5227 \cdot 0.3$) and 67.93% ($0.3774 + 1.0063 \cdot 0.3$) of installed capacity. In other words, these amounts are 145.61 MW and 679.29 MW, respectively. We

use these values in Constraints (21), (25), (72), and (75). After calculating ω_{gt} and ρ_{gt} for each g and t, we replace all numbers less than 0.01 with 0. We do this to remove small coefficients from the model, which enables optimization solvers to perform quick calculations.

We assume that the new build combined cycle gas unit has a minimum power output of 0.2; biomass, coal, and combustion turbine gas units have 0.3; nuclear has 0.4; CCS gas has 0.6. We borrow these numbers from the PowerGenome example input data sets. The minimum output of storage units, solar, wind, and geothermal resources is 0.

5.6 Ramping limits

For reservoir hydro units, we use ramping limits provided by RESOLVE input data [18] and PowerGenome (Table 4). 12.3% and 6.66% in Table 4 come from RESOLVE input data and 8.3% from PowerGenome.

**Zones**	**Up**	**Down**	**Zones**	**Up**	**Down**	**Zones**	**Up**	**Down**
Arizona	0.083	0.083	Colorado	0.083	0.066	Idaho	0.083	0.066
Montana	0.083	0.066	New Mexico	0.083	0.083	Nevada	0.083	0.083
North California	0.123	0.123	Pacific Northwest	0.083	0.066	San Diego	0.123	0.123
South California	0.123	0.123	Utah	0.083	0.066	Wyoming	0.083	0.066

Supplemental Table 4: Ramp-up/down limits as fractions of installed capacity for reservoir hydro units

Table 5 lists ramp-up/down rates for other resources. We borrow these numbers from PowerGenome example input data sets. Ramp-up/down rates are 1 for storage units, geothermal, solar, wind, and run-of-river hydro resources.

**Resources**	**Up**	**Down**	**Resources**	**Up**	**Down**
Biomass	0.60	0.60	Coal	0.25	0.25
Combined cycle gas	0.64	0.64	Steam turbine gas	0.8	0.8
CCS gas	0.64	0.64	Existing combustion turbine gas	0.80	0.80
New combustion turbine gas	0.90	0.90	Nuclear	0.25	0.25
Other peaker	0.90	0.90			

Supplemental Table 5: Ramp-up/down rates as a percent of installed capacity

5.7 Minimum up and down times

Table 6 lists minimum up and down times. We borrow these numbers from [10, supplemental data of GenX model] and PowerGenome example input data sets. Storage units, reservoir hydro, geothermal, solar, wind, and run-of-river hydro resources have minimum up and down times of 0 hours.

**Resources**	**Up**	**Down**	**Resources**	**Up**	**Down**
Biomass	24	24	Coal	24	24
Combined cycle gas	6	6	Existing combustion turbine gas	6	6
New combustion turbine gas	1	1	Steam turbine gas	6	6
CCS gas	6	6	Nuclear	24	24
Other peaker	6	6			

Supplemental Table 6: Minimum up and down times (hours)

5.8 Potentials for new build projects

Geothermal potentials are listed for British Columbia, California, Nevada, Idaho, Oregon, and Utah [19]. We integrate these values for Nevada, Idaho, Oregon, and Utah. For California, we use the potential given by CEC [11] and divide it by three to assign potentials to North California, South California, and San Diego (Table 7).

**Zones**	**Potentials**	**Zones**	**Potentials**	**Zones**	**Potentials**
Idaho	300	Nevada	1,400	North California	671
Pacific Northwest	800	San Diego	671	South California	671
Utah	300				

Supplemental Table 7: Potentials (MW) for new build geothermal resources

For new build reservoir hydro units, we assume that all new resources are non-powered dams. They are existing dams used for non-electricity purposes such as irrigation. It is more efficient and less harmful to the environment to convert those dams into electricity-producing structures than building new stream resources. Their potentials are reported in [20], which we integrate into the model without any alteration except California zones (Table 8). A

single potential is reported for California. We approach detailed data set in supplementary information [21; 20]. We determine potentials in California zones by filtering non-powered dams with states, latitudes, and longitudes.

**Zones**	**Potentials**	**Zones**	**Potentials**	**Zones**	**Potentials**
Arizona	80	Colorado	172	Idaho	12
Montana	88	New Mexico	103	Nevada	16
North California	80.93	Pacific Northwest	201	San Diego	81.56
South California	26.24	Utah	40	Wyoming	45

Supplemental Table 8: Potentials (MW) for new build non-powered dams

For new build hydroelectric pumped storage, we use potentials reported in [19] for all zones, except California zones (Table 9). We benefit from [11] for potentials in California. We assume that California's potential provided by [11] is equally distributed over three zones.

**Zones**	**Potentials**	**Zones**	**Potentials**	**Zones**	**Potentials**
Arizona	4,440	Colorado	790	Idaho	1,150
Montana	1,430	New Mexico	750	Nevada	2,300
North California	1,333	Pacific Northwest	4,898	San Diego	1,333
South California	1,333	Utah	3,100	Wyoming	1,850

Supplemental Table 9: Potentials (MW) for new build hydroelectric pumped storage

For new solar projects, PowerGenome provides utility PV's potential. We use [22] to get potentials of residential PV, commercial PV, and solar thermal with energy storage. Only rooftop solar power potentials are exhibited by [22], but not more disaggregated potentials for residential and commercial PVs. We assume that residential and commercial PV potentials are half rooftop solar power potentials. Having modified the data, we integrate potentials without any alteration for all zones except California zones. We assume California's residential PV, commercial PV, and solar thermal with energy storage potentials are equally distributed over California zones. For Pacific Northwest, the potential is the sum of those of Oregon and Washington (Table 10).

Zones	Residential PV	Commercial PV	Solar Thermal with Energy Storage
Arizona	7,500	7,500	3,528
Colorado	6,000	6,000	3,098
Idaho	1,500	1,500	1,267
Montana	1,000	1,000	557
New Mexico	2,000	2,000	4,860
Nevada	3,500	3,500	2,558
North California	12,666	12,666	908
Pacific Northwest	10,500	10,500	1076
San Diego	12,666	12,666	908
South California	12,666	12,666	908
Utah	3,000	3,000	1,638
Wyoming	500	500	1,956

Supplemental Table 10: Potentials (MW) for new build solar projects

5.9 Net international import for boundary zones

WECC includes areas from U.S., Canada, and Mexico. Since we only focus on the U.S. WECC system in this study, we assume fixed international import/export from Canadian and Mexican WECC systems. Canadian WECC system is connected to the U.S. WECC system through Washington and Montana, and the Mexican WECC system is connected to the U.S. WECC system through California and New Mexico.

International imports/exports by Washington, California, Montana and New Mexico occur within WECC system. Even though Montana and New Mexico have international boundaries with non-WECC electricity systems, these states are not connected to non-WECC electricity systems with transmission lines [23; 24].

We tabulate electricity transfer data provided in [25] and [26] in Table 11. We focus on the most recent data year, 2019 (the data year is 2011 for New Mexico because it does not have international import/export in later years). Notice that export (import) in Transfer by State is the sum of total international export (import) and net interstate export (import) in Total Disposition by State. Net interstate import and export for Montana, Wyoming, Colorado, and New Mexico may also involve transactions with non-WECC states. We assume that

these transactions are negligible.

States	Transfer by State (MW) [25]		Total Disposition by State (MW) [26]			
	Export	Import	Total International Import	Net Interstate Import	Total International Export	Net Interstate Export
Arizona	31,044,814					31,041,999
California	1,956,310	77,460,738	6,672,712	70,788,026	1,956,310	
Colorado		3,420,060		3,420,060		
Idaho		7,567,975		7,567,975		
Montana	11,590,450	70,018	70,018		863,251	10,727,199
New Mexico	13,458,388	44,754	44,754		17,363	13,441,025
Nevada	670,478					670,478
Oregon	8,534,942					8,534,942
Utah	5,551,000					5,551,000
Washington	11,854,170	2,409,006	2,409,006		6,289,765	5,564,405
Wyoming	23,072,845					23,072,845

Supplemental Table 11: Annual electricity transfer data

The difference between total international import and total international export indicates net international import for boundary states. Hence, our modeling zones Pacific Northwest, Montana, San Diego, and New Mexico have hourly net international imports of -443 MW, -90 MW, 538 MW, and 3 MW, respectively (one year has 8,760 hours). We extrapolate these numbers to the modeling year following regionwide demand growth projections. PowerGenome uses a power function and we estimate implied growth rates, see Table 12. Hourly net international imports in 2050 are -597 MW, -122 MW, 717 MW, and 5 MW for Pacific Northwest, Montana, San Diego, and New Mexico, respectively.

Zones	Rate	Zones	Rate	Zones	Rate
Arizona	0.0124	Colorado	0.0122	Idaho	0.0123
Montana	0.0097	New Mexico	0.0124	Nevada	0.0123
North California	0.0097	Pacific Northwest	0.0097	San Diego	0.0093
South California	0.0093	Utah	0.0123	Wyoming	0.0123

Supplemental Table 12: Growth rates to extrapolate net international import

5.10 Fixed import and export for planning reserve

Using the net interstate import and export data provided in Table 11, we calculate fixed import and export for planning reserve by dividing these columns by 8,760 for zones Arizona, Colorado, Idaho, Montana, New Mexico, Nevada, Utah, and Wyoming.

For California zones, we find peak demand in 2050 for North California, South California, and San Diego and scale them, so that sum of peak demands is equal to 1. We multiply hourly net interstate import for California with scaled peak demands to obtain fixed imports for California zones.

For Pacific Northwest, the sum of net interstate exports for Oregon and Washington does not result in Pacific Northwest's export because it overlooks the possible electricity trade between these two states. Ideally, we have to remove this trade amount from the data, but it is unavailable. Alternatively, we assume that the number of transmission lines emanating from Oregon and Washington is scaled with the trade amount. We find that Washington has two 500 kV lines to Idaho and five 500 kV lines to Oregon. Hence, the hourly net interstate export for Washington is multiplied by 2/7 to obtain a rough estimate of fixed export for Oregon. Similarly, Oregon has five 500 kV lines to Washington and three 500 kV lines to other states. Net interstate export for Oregon is multiplied by 3/8 to get a rough estimate of fixed export for Washington. Finally, fixed export for Pacific Northwest is the sum of fixed exports for Oregon and Washington.

To summarize, fixed export and import assumed for planning reserve requirements are reported in Table 13. These are reported for 2050 by using the growth rates reported in Table 12. Our total firm import assumption for WECC is around 9.3 GW, based on the values given Table 11. It is close to 7.7 GW, which is the firm import in 2021 reported in [27].

**Zones**	**Import**	**Export**	**Zones**	**Import**	**Export**
Arizona	0	5,193	Colorado	568	0
Idaho	1,262	0	Montana	0	1,652
New Mexico	0	2,481	Nevada	0	112
North California	5,223	0	Pacific Northwest	0	738
San Diego	970	0	South California	4,637	
Utah	0	926	Wyoming	0	3,848

Supplemental Table 13: Fixed import and export (MW) to meet planning reserve requirement

5.11 Planning reserve margins

We benefit from [19] to integrate planning reserve margins to the model (Table 14). Zonal aggregation in [19] is slightly different than ours. Southwest covers our zones of Arizona and New Mexico; Rocky Mountain covers Colorado and Wyoming; Northwest covers Idaho, Montana, and Pacific Northwest; Basin covers Nevada and Utah; California covers our California zones. We use these margins for BAU and Expanded EIM scenarios. We use California's margin as a region-wide margin in the Regional Market scenario.

**Zones**	**Margin**	**Zones**	**Margin**	**Zones**	**Margin**
Arizona	0.15	Colorado	0.17	Idaho	0.13
Montana	0.13	New Mexico	0.15	Nevada	0.13
North California	0.15	Pacific Northwest	0.13	San Diego	0.15
South California	0.15	Utah	0.13	Wyoming	0.17

Supplemental Table 14: Planning reserve margins

5.12 Operating reserve requirement

Operating reserve requirement is determined as 3% of demand plus 5% of renewable energy generation, also known as 3+5 heuristic rule [28; 29]. We keep the same percentages as a region-wide requirement for Expanded EIM and Regional Market scenarios.

5.13 Proposed transmission projects

We analyze various sources (including [30; 31]) to compile the list of proposed transmission projects (Table 15).

Name	Path	MW	Miles	AC/DC	kV
Centennial West Clean Line	New Mexico to South California	3500	900	HVDC	600
Cross-Tie	Nevada to Utah	1500	213	AC	500
Gateway South	Utah to Wyoming	1500	400	AC	500
Gateway West	Idaho to Wyoming	3000	1000	AC	500
Southline	Arizona to New Mexico	1000	370	AC	345
Southwest Powerlink HVDC Conversion	Arizona to San Diego	1000	165	HVDC	450
SunZia	Arizona to New Mexico	4500	550	AC	500
SWIP North	Idaho to Nevada	2000	275	AC	500
TransWest Express	Nevada to Wyoming	3000	900	AC/HVDC	500
Zephyr	Utah to Wyoming	3000	850	HVDC	500
Boardman - Hemingway	Idaho to Pacific Northwest	2250	290	AC	500
Delaney - Colorado River	Arizona to South California	3200	130		500
Wyoming - Colorado Intertie	Colorado to Wyoming	850	180		345
Great Basin HVDC	Nevada to North California	500	125	HVDC	500
North Gila - Imperial Valley 2	Arizona to San Diego	1250	100	AC	500
Chinook	Montana to Nevada	3000	1000	HVDC	500

Supplemental Table 15: Proposed transmission projects in the west

Our proposed lines are partially harmonious with the CAISO 20-year planning study [32]. There exist some projects which CAISO examines, but we do not consider them in our model. The vice versa is also correct. We list common and divergent projects in our studies in Table 16.

5.14 Hurdle rates costs

CEC itemizes hurdle rate costs (export from zone) for balancing authorities BANC, CAISO, IID, and LADWP in California and for Northwest and Southwest regions of WECC [11]. Northwest overlaps our modeling zones of Colorado, Idaho, Montana, Oregon, Washington, Utah, and Wyoming, and Southwest overlaps Arizona, New Mexico, and Nevada. We integrate the costs for Northwest and Southwest in our data set without any alteration. For California zones, we apply hurdle rate costs given for CAISO. We do not enforce any hurdle rate cost incurred for power flows between North California, South California, and San Diego

CAISO (2022)	Lines Considered in Our Model	Reason for Discrepancy
	North Gila - Imperial Valley 2	
	TransWest Express	
	SWIP North	
	Cross-Tie	
	Sunzia	
	Delaney - Colorado River	
	Southline	
Pacific Transmission Expansion Project		Economic study request
Lucky Corridor		Within NM
GridLiance West		Economic study request
	Boardman - Hemingway	Indirect serve to California
	Gateway South	Indirect serve to California
	Gateway West	Indirect serve to California
	Centennial West Clean Line	Halted, may continue in future
	Zephyr	Early-state
	Wyoming - Colorado Intertie	Early-stage
	Great Basin HVDC	Early-stage
	Chinook	Early-stage
	Southwest Powerlink HVDC Conversion	Conversion

Supplemental Table 16: Proposed projects considered in [32] and our model

(Table 17). Hurdle rate costs are zero for all zones in Expanded EIM and Regional Market scenarios.

Zones	Cost	Zones	Cost	Zones	Cost
Arizona	7.35	Colorado	4.91	Idaho	4.91
Montana	4.91	New Mexico	7.35	Nevada	7.35
North California	10.39	Pacific Northwest	4.91	San Diego	10.39
South California	10.39	Utah	4.91	Wyoming	4.91

Supplemental Table 17: Hurdle rate costs in \$/MWh (export from zones)

5.15 Demand response and involuntary demand curtailment

Our model includes both demand response (voluntary demand curtailment) and involuntary demand curtailment. In demand response, we assume that at most 7.5% of demand can be curtailed with the cost of \$603/MWh. Involuntary demand curtailment charges \$9,000/MWh (value of lost load), and it can curtail demand up to 100% [10].

5.16 RPS and CES targets

We tabulate the most recent state-level RPS and CES targets (Table 18). Oregon has only RPS, and Washington has only CES targets. Hence, we apply both these targets for Pacific Northwest.

All RPS and CES whose target years are earlier than the modeling year are accounted for in the model. Since the modeling year is 2050, RPS and CES, whose target year is closer to 2050, are integrated into the model (e.g., Nevada's second CES target is considered for 2050 because it also satisfies the first one).

**State**	**RPS**	**CES**
Arizona	15% by 2025	100% by 2050
California	60% by 2030	100% by 2045
Colorado	30% by 2020	100% by 2050
Idaho	-	-
Montana	15% by 2015	-
Nevada	-	50% by 2030, 100% by 2050
New Mexico	-	80% by 2040, 100% by 2045
Oregon	25% by 2025, 50% by 2040	-
Utah	20% by 2025	-
Washington	-	15% by 2020, 100% by 2045
Wyoming	-	-

Supplemental Table 18: Clean energy policies

5.17 Retirement ages

PowerGenome retires existing resources and removes them from the generator data set based on two retirement decisions: (i) Resources are already planned for retirement in reality, (ii) users assign retirement ages for resources. We assign the following retirement ages: Wind has 30 years; coal and nuclear have 60 years; geothermal has 100 years; reservoir hydro, run-of-river hydro, and hydroelectric pumped storage have 500 years; and all other resources have 40 years.

5.18 Costs of transmission investments

Reinforcement in transmission lines charges $3,037 per MW.mile in North and South California (for all lines ending in either of these zones) and $1,350 per MW.mile in other zones. Considering that is 6.9% and the expected service life is 60 years, we calculate the capital recovery factor as 7.26%. Hence, the annual charge is $98 per MW.mile in North and South California, and $159.25 per MW.mile in other zones [10, GenX supplementary information].

5.19 Other inputs

We assume that fixed O&M costs for transmission lines are 1/20 of the reinforcement cost.

We assume that initial and final energy levels of storage units and reservoir hydro resources are half of the full energy capacity.

Reservoir hydro capacities in our data set are 8.7, 2.7, and 35.6 GW for California, Arizona/New Mexico, and the rest of the zones, respectively. These numbers are reported in intervals of 7.6-13.9, 2.7-3.5, and 21.5-417 GW, respectively in [27] (considered with derating rates).

Colstrip Transmission System plans to update the transmission line east of Garrison substation, which affects transmission capacity between Montana and Pacific Northwest [33]. The incremental capacity is reported to be 800 MW, and this additional capacity will be available after 2022. We make this update in our data.

We assume that the minimum reservoir energy level is 5% of energy capacity for reservoir hydro resources.

Our initial set of generators included many thermal units. Since these units are tied to integer variables, solving the resulting model is cumbersome. We remove all biomass units whose cluster size is less than 50 MW. By doing this, we do not sacrifice the solution quality but save enormous time in computation. At the end, we have 40 clusters for existing thermal resources.

We copy utility PV capacity factors for commercial and residential PV. We also copy the

existing reservoir hydro capacity factor for new build resources.

PowerGenome settings file enables users to adjust the number of clusters for off-shore wind, land-based wind, and utility PV for each zone separately. This adjustment is possible by enforcing thresholds for minimum capacity, maximum Levelized cost of electricity, and the maximum number of clusters. Our experience shows that tweaking these parameters does not help get the ideal set of clusters. We devise our own approach:

1. First, run PowerGenome without enforcing any threshold in its settings. This gives us all possible clusters.
2. Remove all clusters with a capacity of less than 50 MW.
3. Calculate the weighted average capacity factor for clusters/resources.

For each off-shore wind, land-based wind, and utility PV and each zone separately:

1. Order all clusters in descending order of weighted average capacity factor.
2. Starting from the first cluster, accept all until one of the following stopping conditions:
   - Cluster's weighted average capacity factor is less than 0.26 for land-based and off-shore wind and 0.16 for utility PV.
   - Cumulative sum of potentials is larger than 50,000 MW for land-based and off-shore wind and 100,000 MW for utility PV.
   - Number of accepted clusters is larger than 100.

We assume in Expanded EIM scenario that fixed import/export to meet planning reserve requirements are 40% higher than the BAU scenario. 40% increase is the maximum elevation for which resulting fixed import/export is still less than transmission line capacities, including existing and proposed lines.

In-state requirement for RPS and CES targets is 75% [34].

6 Sensitivity analysis

In this section, we clarify the sensitivity analysis for in-state RPS/CES ratios, operating reserve requirements, and hurdle rates.

6.1 In-state CES/RPS ratios

In the baseline, we assume that 75% of the clean generation should be delivered from in-state clean resources to meet CES/RPS targets under the State Policy scenario [34]. In other words, the remaining 25% of the clean generation can be supplied using out-of-state Renewable Energy Credits (RECs). In addition, we examine how varying in-state CES/RPS ratios will impact our findings—0%, 25%, 50%, 75% (baseline), 87.5%, and 100%, similar to the approach in [34]. If in-state CES/RPS ratios are 100%, this corresponds to the least flexible case in terms of REC trading.

6.2 Operating reserve requirements

In the baseline, we adopt the "3+5" heuristic, meaning that operating reserves (both up and down) have to be no less than 3% of the load and 5% of the renewable output at each hour, as described in the Western Wind and Solar Integration Study and other modeling studies [28; 29].

The exact operating reserve requirements in practice vary by product and market. The reserve requirement levels are calculated by [35] based on methods from the Western Electricity Coordinating Council Transmission Expansion Planning Policy Committee (WECC TEPPC) [36]. In the report, spinning reserves have to be no less than 3% of the load, and ramping reserves have to be no less than 10% of wind generation and 4% of solar generation.

Due to the uncertain and variable nature of wind and solar resources, high VRE penetrations lead to increases in reserves that are necessary for the power systems [36]. Hence, we examine higher levels of operating reserve requirements under all the regionalization and policy scenarios: (1) 3% of the load and 5% of the renewable output (baseline), (2) 3% of the load and 10% of the renewable output, and (3) 5% of the load and 10% of the renewable output.

6.3 Hurdle rates

We use hurdle rates as a proxy for the inter-jurisdictional transaction barriers. The hurdle rate costs effectively increase the trading costs between two non-coordinating zones. The baseline cost assumptions are obtained from the CEC SB100 Joint Agency Report [11]. Furthermore, we consider two additional sets of hurdle rates: (1) alternative rates, and (2) low rates. We obtain the alternative rates based on the CPUC System Reliability Modeling Datasets [37], and the low rates by discounting the baseline rates by 50%. We summarize hurdle rates in Table 19.

**Zones**	**Baseline (CEC)**	**Alternative rates (CPUC)**	**Low rates (CEC low)**
Arizona	7.35	4.622	3.675
Colorado	4.91	5.827	2.455
Idaho	4.91	3.124	2.455
Montana	4.91	5.546	2.455
New Mexico	7.35	4.867	3.675
Nevada	7.35	8.143	3.675
North California	10.39	12.694	5.195
Pacific Northwest	4.91	3.604	2.455
San Diego	10.39	12.694	5.195
South California	10.39	12.694	5.195
Utah	4.91	3.604	2.455
Wyoming	4.91	5.827	2.455

Supplemental Table 19: Hurdle rates used in sensitivity analysis

We summarize the design of sensitivity analysis in Table 20, followed by key results including system costs (Table 21, Table 22, and Table 23), solar capacities (Table 24, Table 25, and Table 26), wind capacities (Table 27, Table 28, and Table 29), storage capacities (Table 30, Table 31, and Table 32), transmission capacities (Table 33, Table 34, and Table 35), low-carbon firm capacities (Table 36, Table 37, and Table 38), and carbon-emitting firm capacities (Table 39, Table 40, and Table 41). Column headings in bold refer to baseline settings in these tables.

	Sensitivity factors		
	**In-state CES/RPS ratios**	**Operating reserve requirements**	**Hurdle rates**
**State Policy**			
Inc. Coordination	0%, 25%, 50%, **75%**, 87.5%, 100%	**3%+5%**, 3%+10%, 5%+10%	**CEC**, CEC low, CPUC high
BAU	0%, 25%, 50%, **75%**, 87.5%, 100%	**3%+5%**, 3%+10%, 5%+10%	**CEC**, CEC low, CPUC high
Expanded EIM	0%, 25%, 50%, **75%**, 87.5%, 100%	**3%+5%**, 3%+10%, 5%+10%	N/A
Regional Market	0%, 25%, 50%, **75%**, 87.5%, 100%	**3%+5%**, 3%+10%, 5%+10%	N/A
Full Coordination	N/A	**3%+5%**, 3%+10%, 5%+10%	N/A
**Regional 100% CES**			
BAU	N/A	**3%+5%**, 3%+10%, 5%+10%	**CEC**, CEC low, CPUC high
Expanded EIM	N/A	**3%+5%**, 3%+10%, 5%+10%	N/A
Regional Market	N/A	**3%+5%**, 3%+10%, 5%+10%	N/A
**Count**	**24 cases**	**24 cases**	**9 cases**

Supplemental Table 20: Summary table for sensitivity analysis. N/A indicates that the sensitivity factors are not applied to these scenarios. Records in bold refers to baseline settings.

7 Justification of Continued State Policy

Continued State Policy scenario is more optimistic than the current clean energy goals of the states. As described in the paper, if the major utilities have 100% CES targets (or a similar goal) in a state or if other clean enery goals of the state lead to a de facto CES, then we assume 100% CES goal for that state. In other words, we assign 100% CES targets to states being on track to do so.

We decide to implement 100% CES targets for Arizona, Colorado, and California for the

**In-state CES/RPS ratios**	0%	25%	50%	**75%**	87.5%	100%
**State Policy Scenario**						
Incomplete Coordination	38.95	38.95	38.95	38.96	38.97	38.97
BAU	36.56	36.59	36.66	36.73	36.76	36.79
Expanded EIM	35.96	35.98	36.05	36.12	36.15	36.18
Regional Market	35.71	35.73	35.80	35.88	35.91	35.95

Supplemental Table 21: System costs (B$/year) under different in-state CES/RPS ratios.

**Operating reserve requirements**	**3%+5%**	3%+10%	5%+10%
**State Policy Scenario**			
Incomplete Coordination	38.96	38.99	39.03
BAU	36.73	36.77	36.82
Expanded EIM	36.12	36.12	36.14
Regional Market	35.88	35.88	35.93
Full Coordination	35.71	35.71	35.76
**Regional 100% CES Scenario**			
BAU	41.98	42.00	42.05
Expanded EIM	41.65	41.66	41.69
Regional Market	41.61	41.62	41.66

Supplemental Table 22: System costs (B$/year) under different reserve requirements.

following reasons.

Even though the Arizona Corporation Commission rejected a 100% CES by 2050 in 2021, there was much discussion about it. At the same time, there have been pledges from the three main utility companies: Arizona's largest utility, APS (∼1.3m households), announced 100% CES by 2050, Tucson Electric Power (∼0.4m households) plans to reduce carbon emissions by 80% by 2035, and have a 70% RPS by 2035, and Salt River Project (∼1.1m customers) plans to reduce carbon by 65% in 2035 and 90% by 2050. Hence, we think a continuation of these pledges will work de facto similar to a 100% CES which would be codified by the state.

Colorado SB19-263 requires 100% CES by 2050 for utilities serving 500,000 or more customers, which then only applies to Xcel (which takes up ∼60% of the state's load) [38].

**Hurdle rates**	**CEC**	CEC low	CUPC high
**State Policy Scenario**			
Incomplete Coordination	38.96	38.86	38.95
BAU	36.73	36.55	36.69
**Regional 100% CES Scenario**			
BAU	41.98	41.90	41.97

Supplemental Table 23: System costs (B$/year) under different hurdle rates.

**In-state CES/RPS ratios**	0%	25%	50%	**75%**	87.5%	100%
**State Policy Scenario**						
Incomplete Coordination	168.21	168.21	168.15	168.33	168.37	168.37
BAU	140.68	140.98	141.94	142.24	142.24	142.51
Expanded EIM	141.48	141.43	141.91	142.99	143.05	143.50
Regional Market	136.83	137.96	137.95	138.96	139.05	138.74

Supplemental Table 24: Solar capacities (GW) under different in-state CES/RPS ratios.

However, Colorado also has an SB16 that requires the whole economy to reduce greenhouse gas emissions by 100% by 2050. Hence, we think it is reasonable to assume that the power sector has a de facto 100% CES target [39].

Regarding California, the net-zero target set for 2045 applies to all activities and sectors within the state (e.g., electricity production, transportation, buildings, agriculture, etc.). Federal entities within the state does not need to follow the target. However, energy consumption by federal entities accounted for 2% of total energy consumption in the state in 2000 [40].

**Operating reserve requirements**	**3%+5%**	3%+10%	5%+10%
**State Policy Scenario**			
Incomplete Coordination	168.33	168.00	168.11
BAU	142.24	141.73	141.75
Expanded EIM	142.99	143.06	143.24
Regional Market	138.96	139.13	139.95
Full Coordination	136.83	136.85	136.86
**Regional 100% CES Scenario**			
BAU	176.07	175.84	175.68
Expanded EIM	175.46	174.88	173.99
Regional Market	175.47	175.63	175.80

Supplemental Table 25: Solar capacities (GW) under different reserve requirements.

**Hurdle rates**	**CEC**	CEC low	CUPC high
**State Policy Scenario**			
Incomplete Coordination	168.33	168.45	168.38
BAU	142.24	142.65	142.02
**Regional 100% CES Scenario**			
BAU	176.07	174.46	175.65

Supplemental Table 26: Solar capacities (GW) under different hurdle rates.

**In-state CES/RPS ratios**	0%	25%	50%	**75%**	87.5%	100%
**State Policy Scenario**						
Incomplete Coordination	44.66	44.66	44.59	44.58	44.59	44.59
BAU	27.11	29.08	32.51	34.31	34.43	34.31
Expanded EIM	25.21	27.12	30.03	31.62	32.03	31.79
Regional Market	23.70	24.01	27.39	29.49	30.09	30.58

Supplemental Table 27: Wind capacities (GW) under different in-state CES/RPS ratios.

**Operating reserve requirements**	**3%+5%**	3%+10%	5%+10%
**State Policy Scenario**			
Incomplete Coordination	44.58	44.31	44.65
BAU	34.31	33.41	33.44
Expanded EIM	31.62	31.46	31.29
Regional Market	29.49	28.65	27.61
Full Coordination	23.70	23.47	23.46
**Regional 100% CES Scenario**			
BAU	59.40	58.90	58.81
Expanded EIM	60.16	60.07	60.53
Regional Market	62.97	62.36	62.24

Supplemental Table 28: Wind capacities (GW) under different reserve requirements.

**Hurdle rates**	**CEC**	CEC low	CUPC high
**State Policy Scenario**			
Incomplete Coordination	44.58	45.40	44.86
BAU	34.31	33.44	33.62
**Regional 100% CES Scenario**			
BAU	59.40	59.93	60.00

Supplemental Table 29: Wind capacities (GW) under different hurdle rates.

**In-state CES/RPS ratios**	0%	25%	50%	**75%**	87.5%	100%
**State Policy Scenario**						
Incomplete Coordination	81.87	81.87	81.80	81.77	81.74	81.71
BAU	79.27	79.22	79.48	79.38	79.31	78.46
Expanded EIM	73.47	73.52	74.08	73.22	71.27	70.50
Regional Market	53.05	53.10	52.97	53.05	52.99	53.05

Supplemental Table 30: Storage capacities (GW) under different in-state CES/RPS ratios.

**Operating reserve requirements**	**3%+5%**	3%+10%	5%+10%
**State Policy Scenario**			
Incomplete Coordination	81.77	82.70	86.28
BAU	79.38	81.27	83.97
Expanded EIM	73.22	73.15	73.67
Regional Market	53.05	53.87	57.77
Full Coordination	53.05	53.82	57.55
**Regional 100% CES Scenario**			
BAU	86.85	87.62	90.21
Expanded EIM	79.88	80.69	83.18
Regional Market	76.56	77.25	80.61

Supplemental Table 31: Storage capacities (GW) under different reserve requirements.

**Hurdle rates**	**CEC**	CEC low	CUPC high
**State Policy Scenario**			
Incomplete Coordination	81.77	81.87	81.72
BAU	79.38	80.05	79.29
**Regional 100% CES Scenario**			
BAU	86.85	86.33	86.51

Supplemental Table 32: Storage capacities (GW) under different hurdle rates.

**In-state CES/RPS ratios**	0%	25%	50%	**75%**	87.5%	100%
**State Policy Scenario**						
Incomplete Coordination	58.21	58.21	58.22	58.22	58.24	58.25
BAU	104.33	103.80	103.09	102.94	103.59	104.08
Expanded EIM	108.19	107.09	106.76	107.23	107.08	107.20
Regional Market	109.54	109.30	108.79	108.51	108.56	108.45

Supplemental Table 33: Transmission capacities (GW) under different in-state CES/RPS ratios.

**Operating reserve requirements**	**3%+5%**	3%+10%	5%+10%
**State Policy Scenario**			
Incomplete Coordination	58.22	58.04	58.13
BAU	102.94	103.30	103.32
Expanded EIM	107.23	107.34	106.84
Regional Market	108.51	108.67	108.62
Full Coordination	109.54	109.56	109.16
**Regional 100% CES Scenario**			
BAU	51.56	51.33	51.31
Expanded EIM	52.83	52.85	53.33
Regional Market	55.77	55.42	55.38

Supplemental Table 34: Transmission capacities (GW) under different reserve requirements.

**Hurdle rates**	**CEC**	CEC low	CUPC high
**State Policy Scenario**			
Incomplete Coordination	58.22	58.77	58.52
BAU	102.94	103.93	102.89
**Regional 100% CES Scenario**			
BAU	51.56	52.13	51.94

Supplemental Table 35: Transmission capacities (GW) under different hurdle rates.

**In-state CES/RPS ratios**	0%	25%	50%	**75%**	87.5%	100%
**State Policy Scenario**						
Incomplete Coordination	68.78	68.78	68.78	68.75	68.76	68.77
BAU	53.33	53.33	53.33	53.33	53.33	53.33
Expanded EIM	53.33	53.33	53.33	53.33	53.33	53.33
Regional Market	53.33	53.33	53.33	53.33	53.33	53.33

Supplemental Table 36: Low-carbon firm capacities (GW) under different in-state CES/RPS ratios.

**Operating reserve requirements**	**3%+5%**	3%+10%	5%+10%
**State Policy Scenario**			
Incomplete Coordination	68.75	68.91	68.81
BAU	53.33	53.33	53.33
Expanded EIM	53.33	53.33	53.33
Regional Market	53.33	53.33	53.33
Full Coordination	53.33	53.33	53.33
**Regional 100% CES Scenario**			
BAU	89.56	89.80	89.86
Expanded EIM	89.73	89.84	89.84
Regional Market	87.95	88.06	88.06

Supplemental Table 37: Low-carbon firm capacities (GW) under different reserve requirements.

**Hurdle rates**	**CEC**	CEC low	CUPC high
**State Policy Scenario**			
Incomplete Coordination	68.75	68.41	68.62
BAU	53.33	53.33	53.33
**Regional 100% CES Scenario**			
BAU	89.56	89.84	89.43

Supplemental Table 38: Low-carbon firm capacities (GW) under different hurdle rates.

**In-state CES/RPS ratios**	0%	25%	50%	**75%**	87.5%	100%
**State Policy Scenario**						
Incomplete Coordination	30.19	30.19	30.23	30.27	30.27	30.26
BAU	64.53	63.81	62.52	62.04	61.94	61.82
Expanded EIM	64.94	64.37	63.49	62.99	62.79	62.87
Regional Market	66.96	66.79	65.69	65.02	64.89	64.71

Supplemental Table 39: Carbon-emitting firm capacities (GW) under different in-state CES/RPS ratios.

**Operating reserve requirements**	**3%+5%**	3%+10%	5%+10%
**State Policy Scenario**			
Incomplete Coordination	30.27	30.17	30.03
BAU	62.04	62.32	62.22
Expanded EIM	62.99	63.08	63.13
Regional Market	65.02	65.29	65.44
Full Coordination	66.96	67.04	66.98
**Regional 100% CES Scenario**			
BAU	0.00	0.00	0.00
Expanded EIM	0.00	0.00	0.00
Regional Market	0.00	0.00	0.00

Supplemental Table 40: Carbon-emitting firm capacities (GW) under different reserve requirements.

**Hurdle rates**	**CEC**	CEC low	CUPC high
**State Policy Scenario**			
Incomplete Coordination	30.27	30.35	30.31
BAU	62.04	62.09	62.28
**Regional 100% CES Scenario**			
BAU	0.00	0.00	0.00

Supplemental Table 41: Carbon-emitting firm capacities (GW) under different hurdle rates.

8 Additional result figures

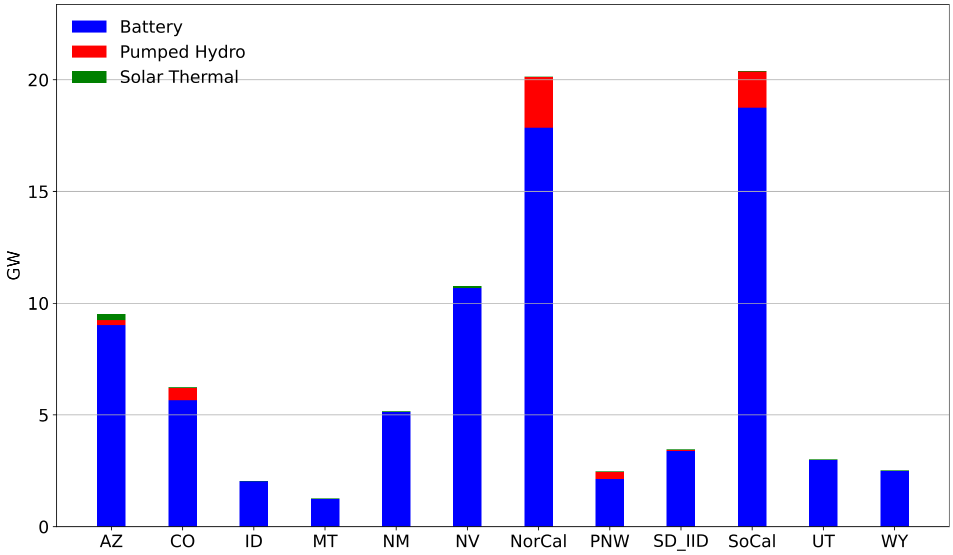

Supplemental Figure 1: Capacities for 2050 (BAU + Regional 100% CES).

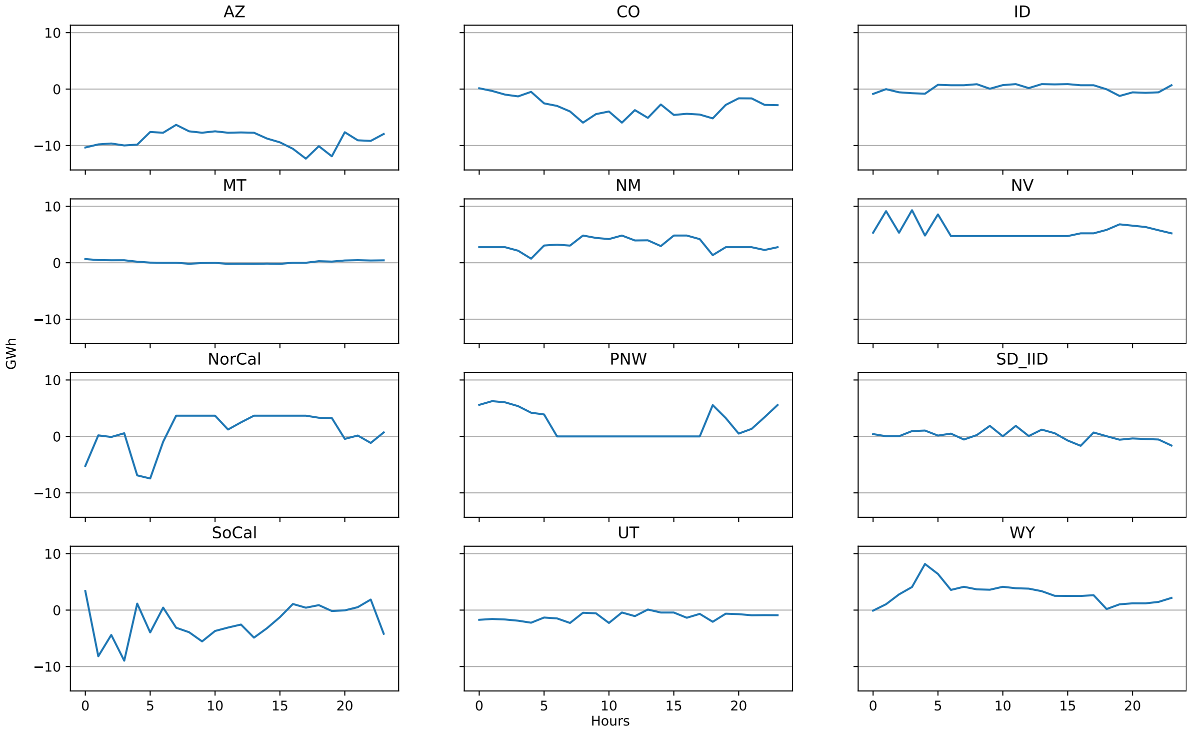

Supplemental Figure 2: Net export in a typical day of 2050 for BAU + Regional 100% CES scenario. Positive (negative) numbers represent export (import). Each panel represents one modeling zone.

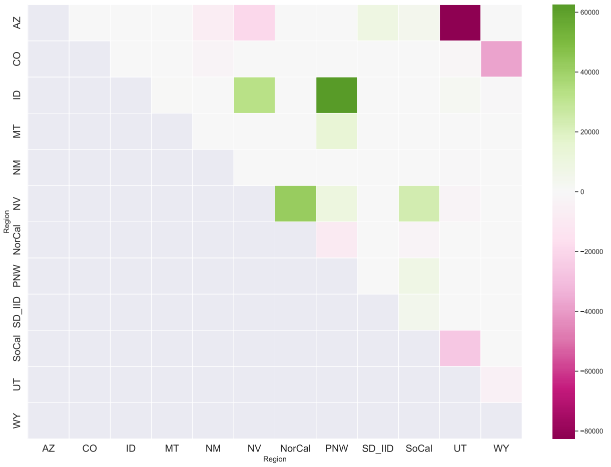

(a) BAU + State Policy

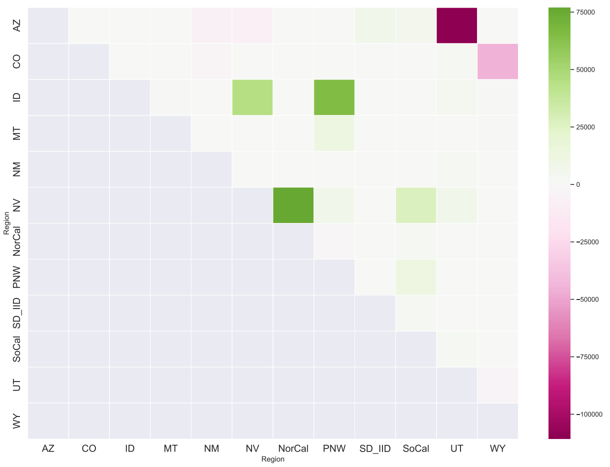

(b) Expanded EIM + State Policy

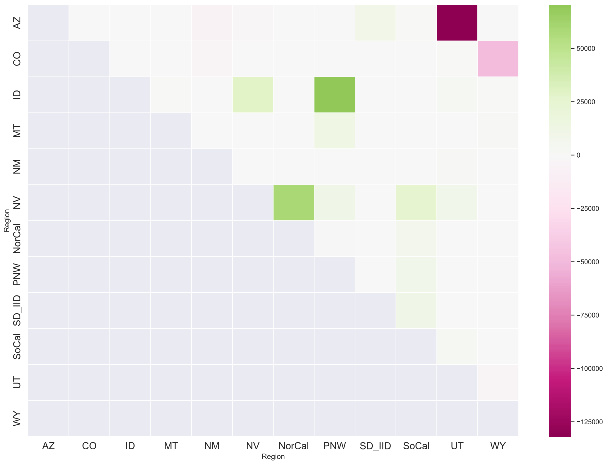

(c) Regional Market + State Policy

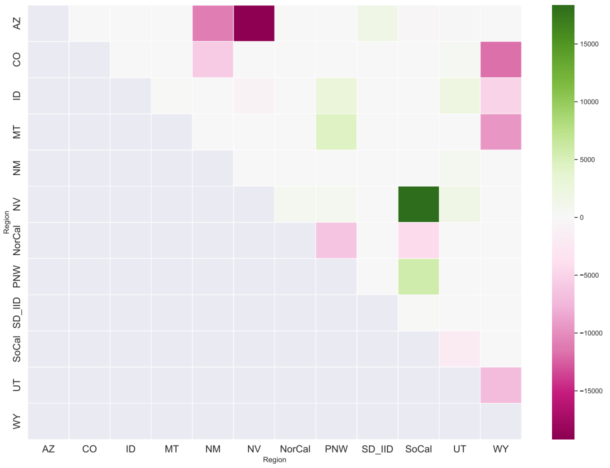

(d) BAU + Regional 100% CES

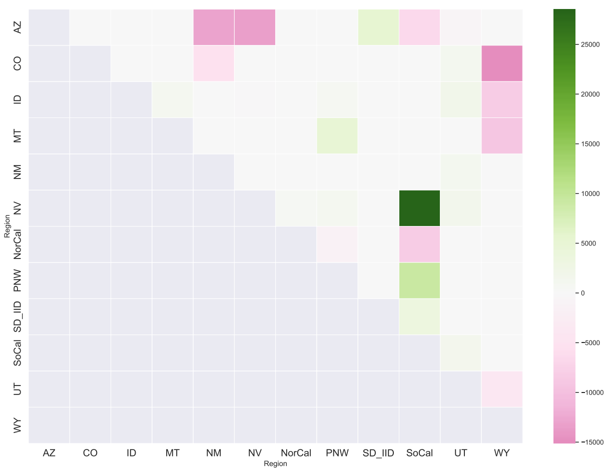

(e) Expanded EIM + Regional 100% CES

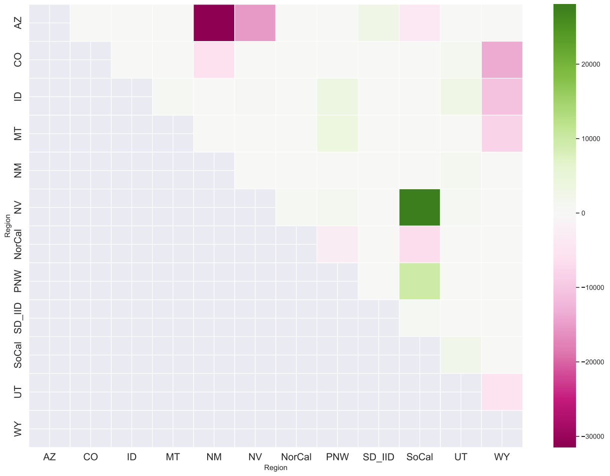

(f) Regional Market + Regional 100% CES

Supplemental Figure 3: Power transfer between zones for 2050. Positive (negative) number indicates export (import). Each panel represents one scenario.

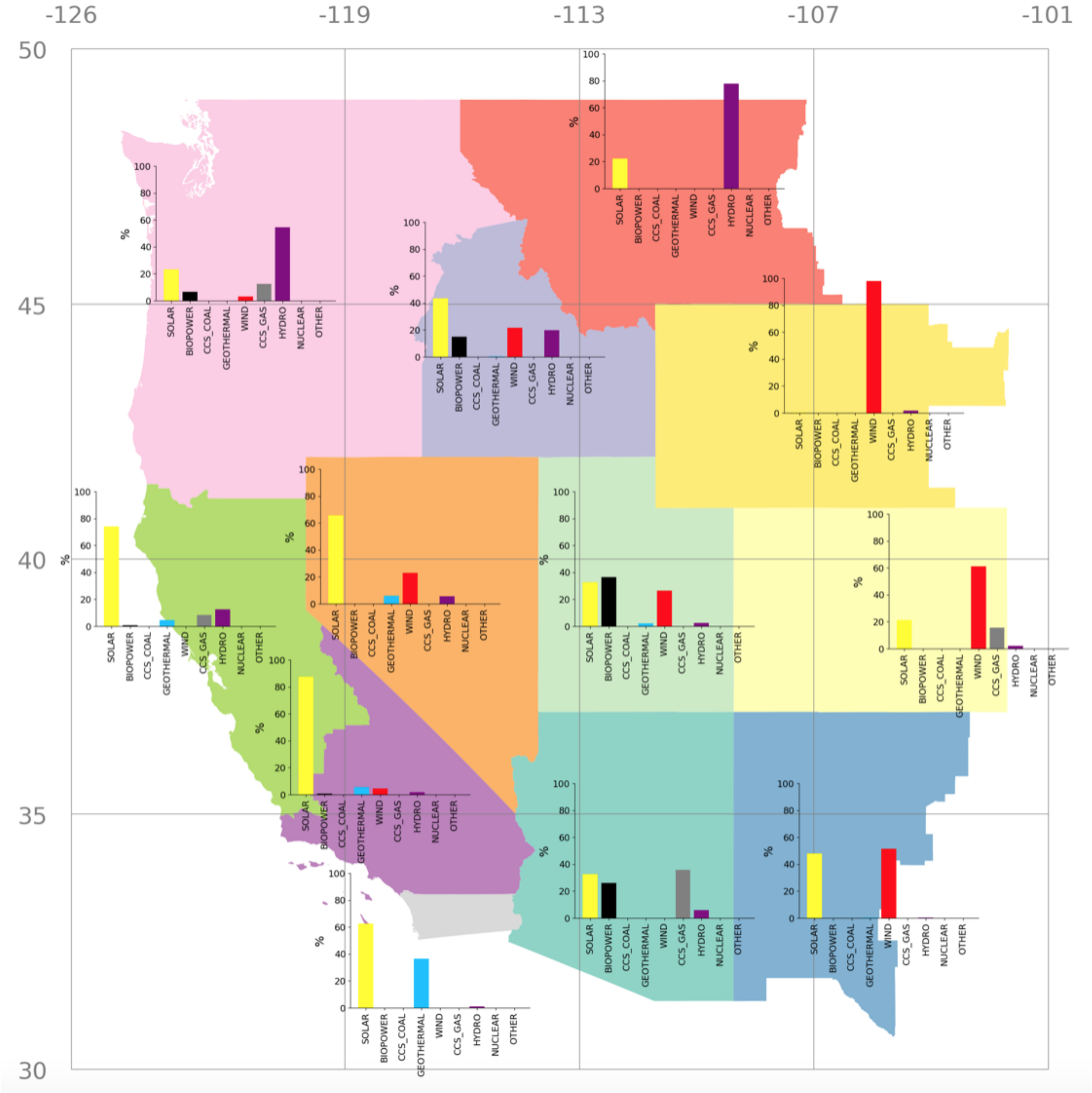

Supplemental Figure 4: Dispatch share for 2050 (BAU + Regional 100% CES). Straight gray lines are longitudes and latitudes. Source: U.S. EPA [9]

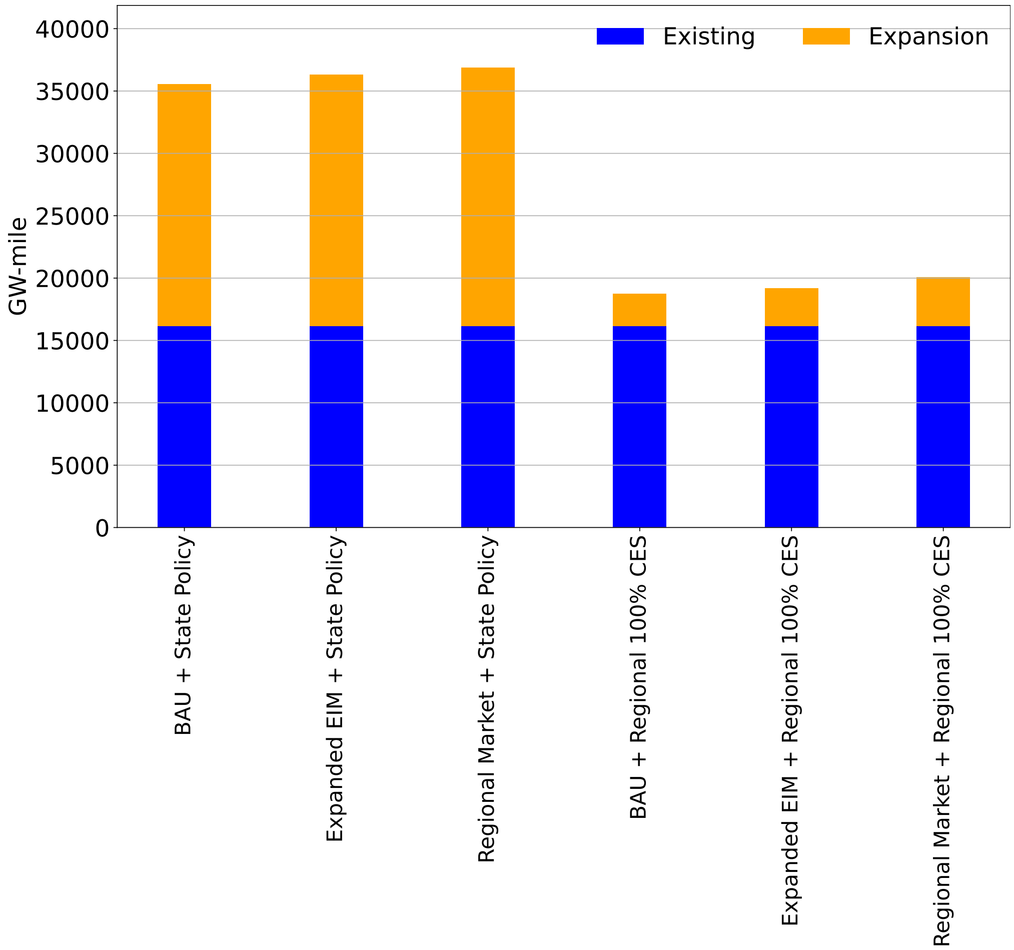

Supplemental Figure 5: GW-mile of transmission lines for 2050

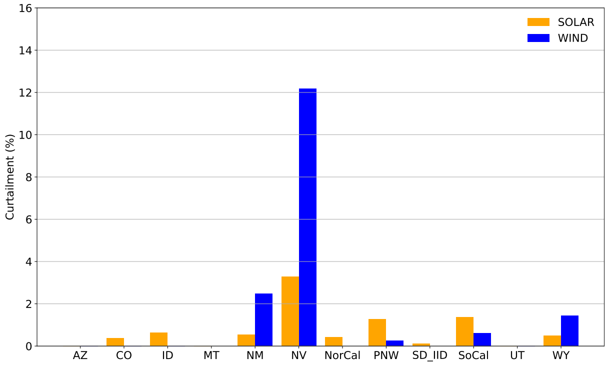

(a) BAU + Regional 100% CES

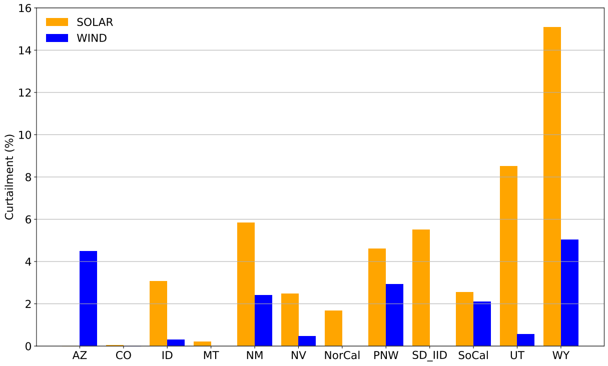

(b) Regional Market + Regional 100% CES

Supplemental Figure 6: Curtailment in 2050 for BAU + Regional 100% CES scenario (left) and Regional Market + Regional 100% CES scenario (right).

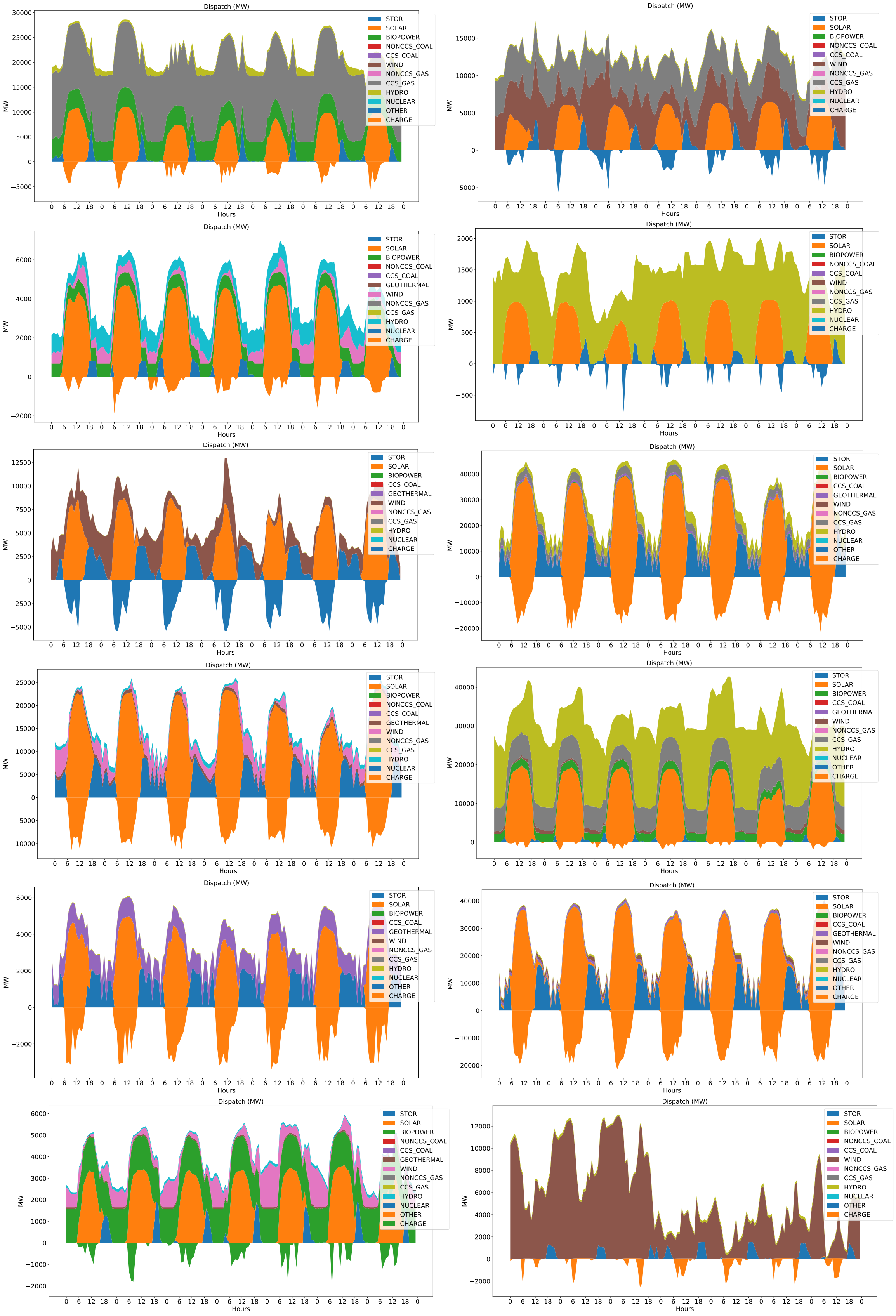

Supplemental Figure 7: Dispatch in peak week of 2050 (BAU + Regional 100% CES) (from left to right and from above to bottom for AZ, CO, ID, MT, NM, NorCal, NV, PNW, SD_IID, SoCal, UT, and WY)

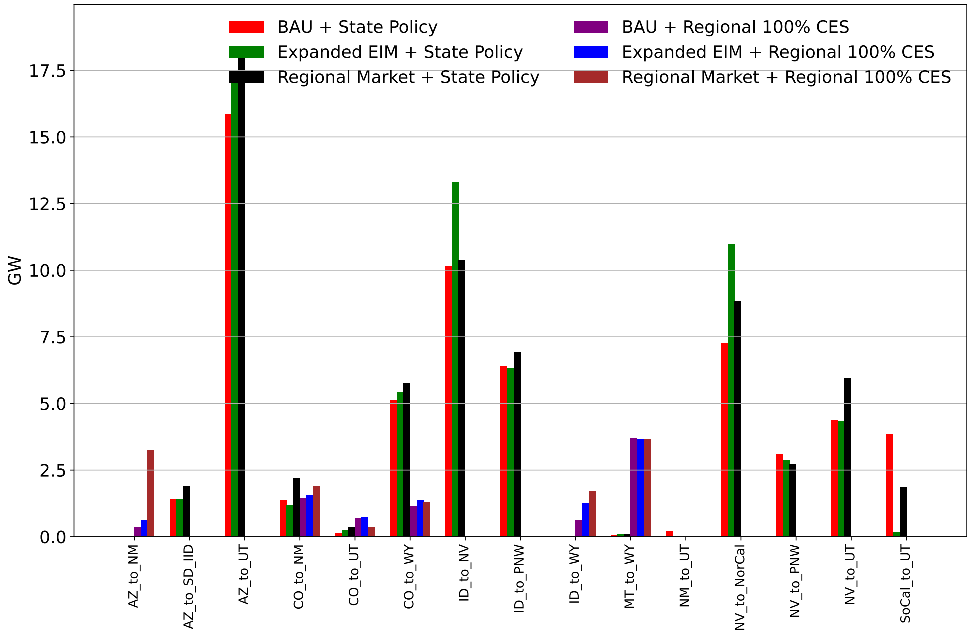

Supplemental Figure 8: Transmission capacity expansions over all available paths with different scenarios.

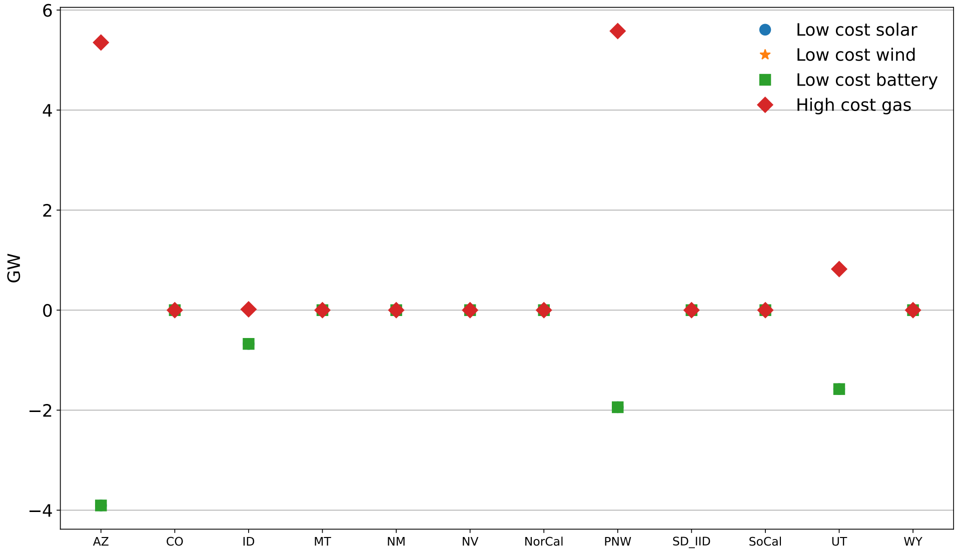

Supplemental Figure 9: Change in capacity of biomass from reference cost case for 2050 (BAU + Regional 100% CES)

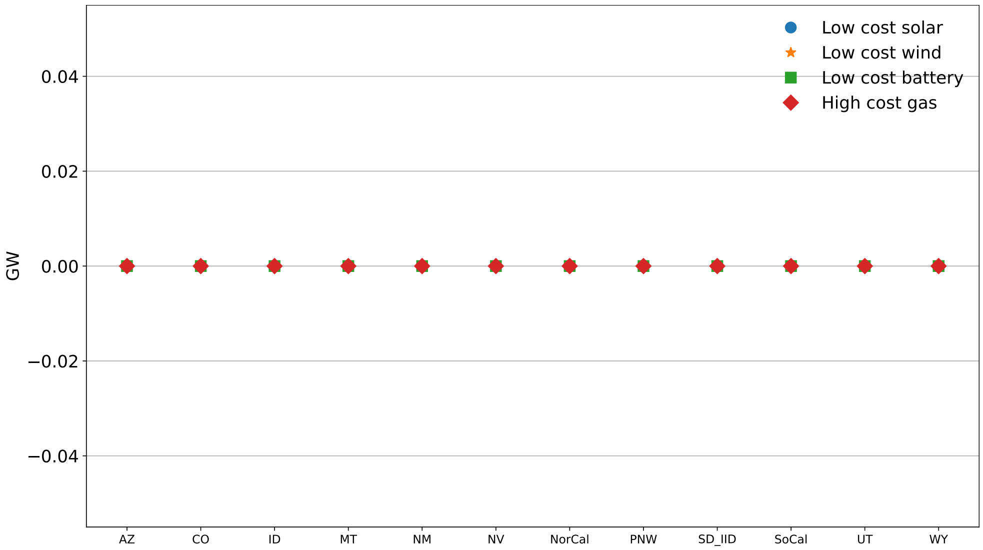

Supplemental Figure 10: Change in capacity of CCS coal from reference cost case for 2050 (BAU + Regional 100% CES)

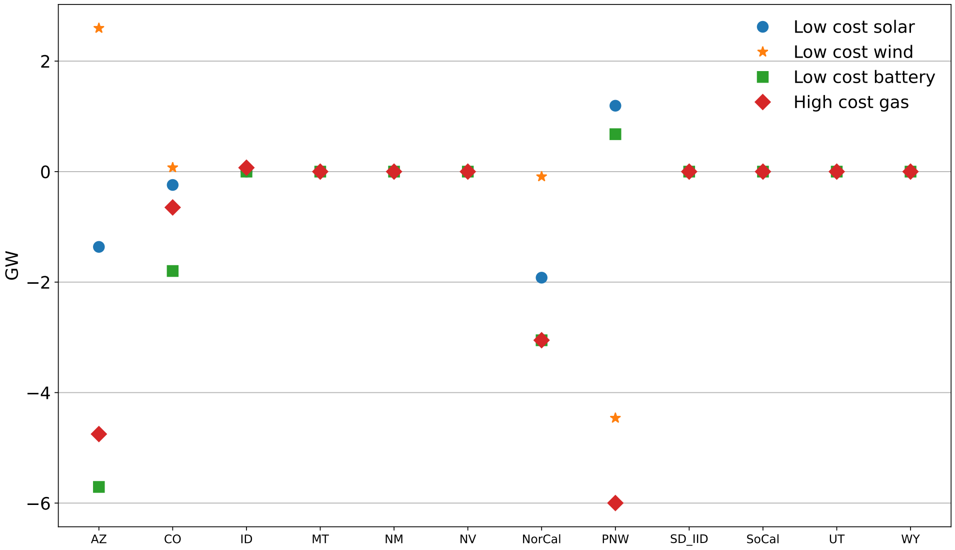

Supplemental Figure 11: Change in capacity of CCS gas from reference cost case for 2050 (BAU + Regional 100% CES)

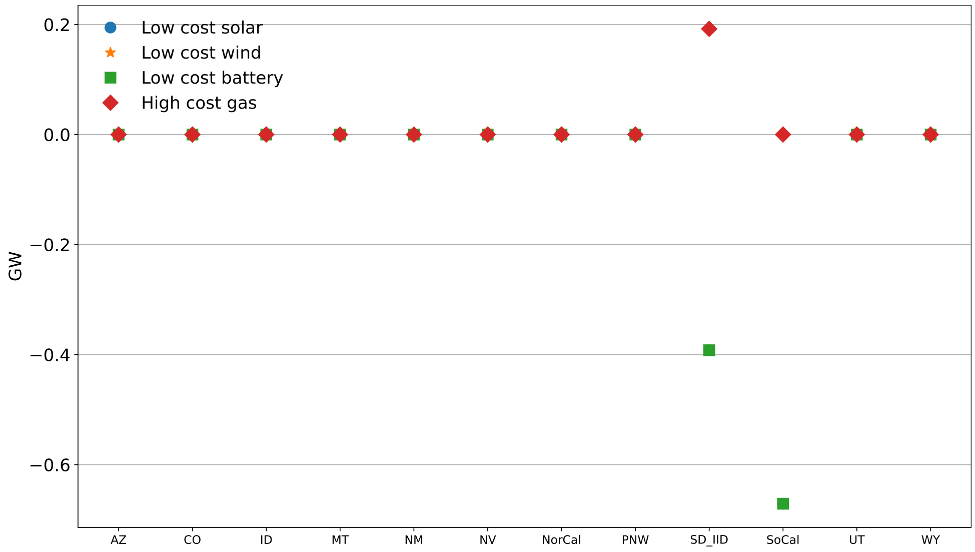

Supplemental Figure 12: Change in capacity of geothermal from reference cost case for 2050 (BAU + Regional 100% CES)

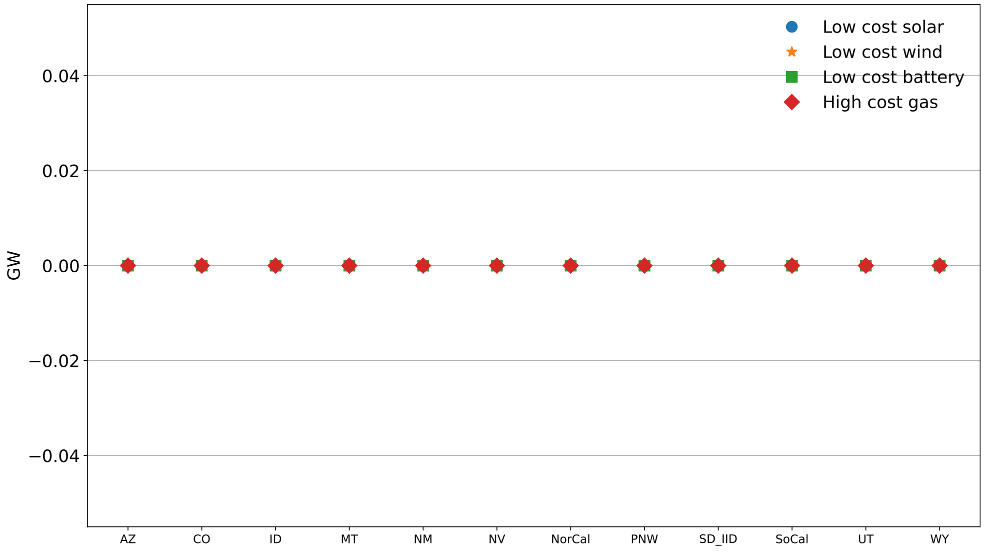

Supplemental Figure 13: Change in capacity of reservoir hydro from reference cost case for 2050 (BAU + Regional 100% CES)

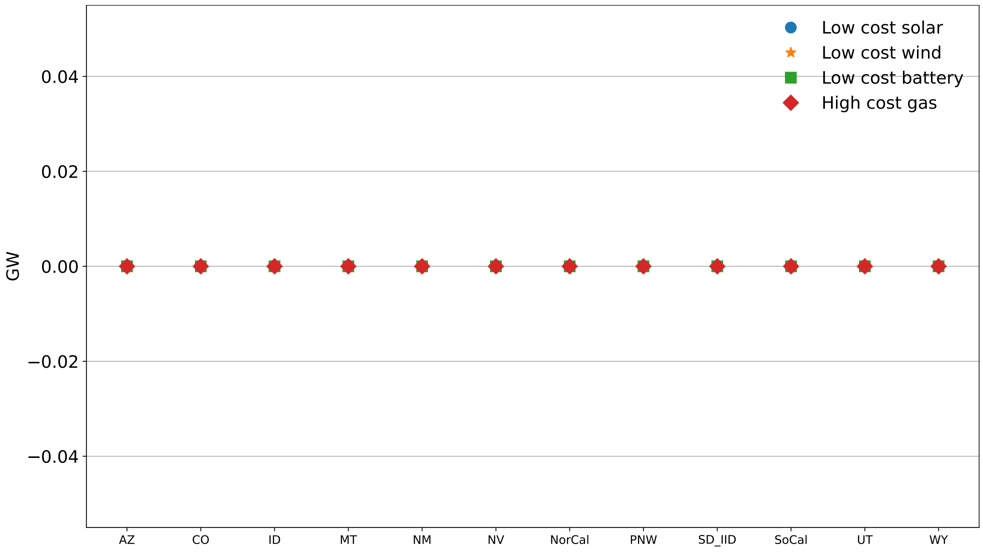

Supplemental Figure 14: Change in capacity of non-CCS coal from reference cost case for 2050 (BAU + Regional 100% CES)

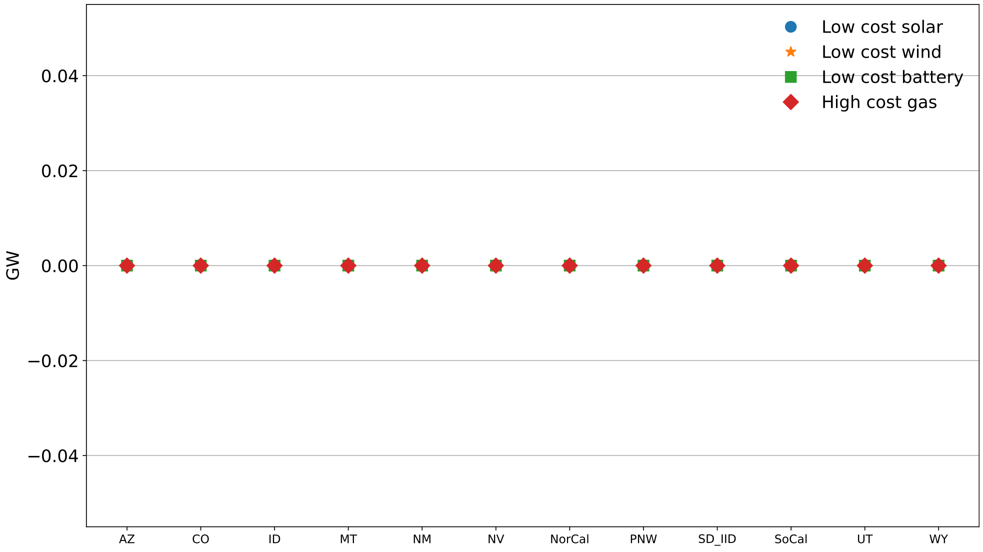

Supplemental Figure 15: Change in capacity of non-CCS gas from reference cost case for 2050 (BAU + Regional 100% CES)

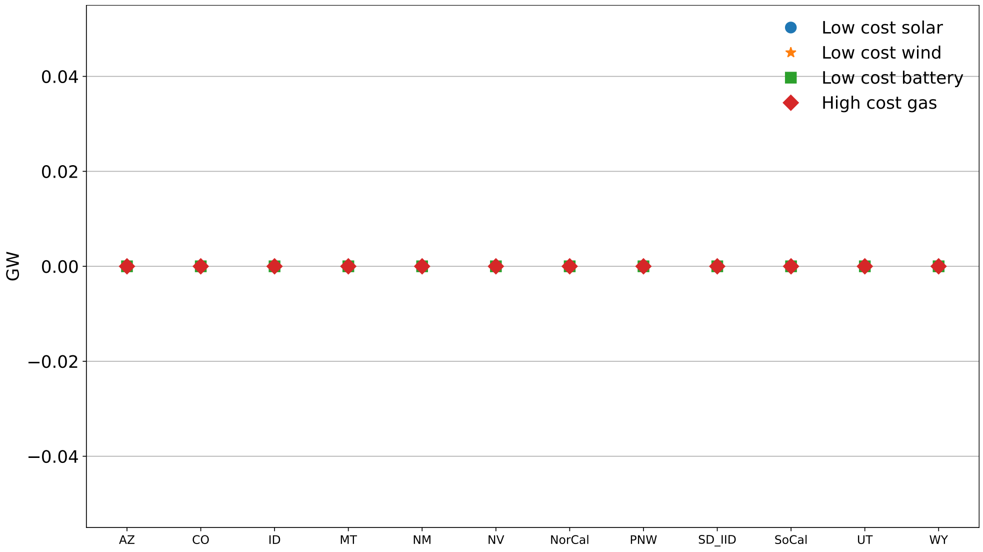

Supplemental Figure 16: Change in capacity of nuclear from reference cost case for 2050 (BAU + Regional 100% CES)

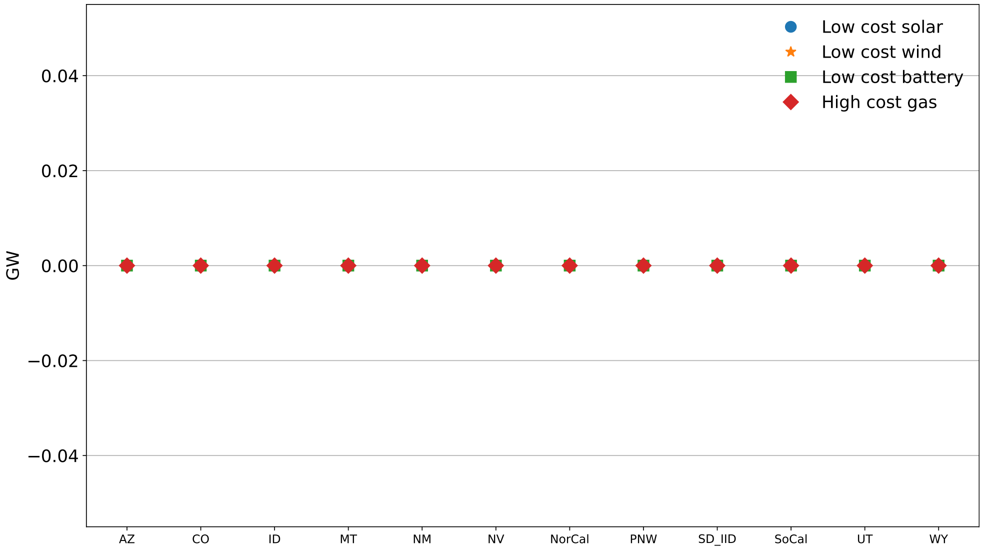

Supplemental Figure 17: Change in capacity of other resources from reference cost case for 2050 (BAU + Regional 100% CES)

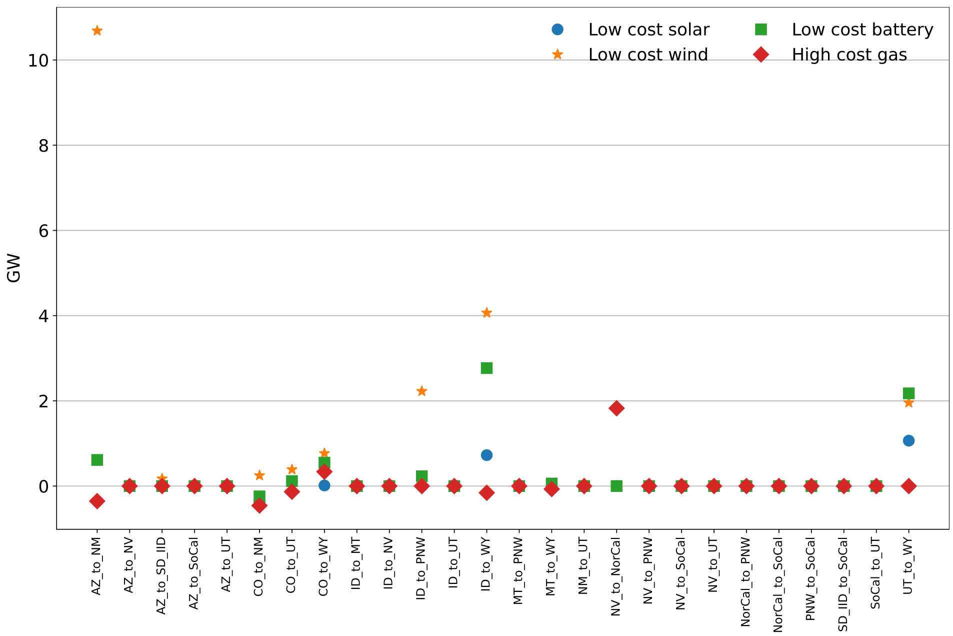

Supplemental Figure 18: Change in capacity of paths from reference cost case for 2050 (BAU + Regional 100% CES)

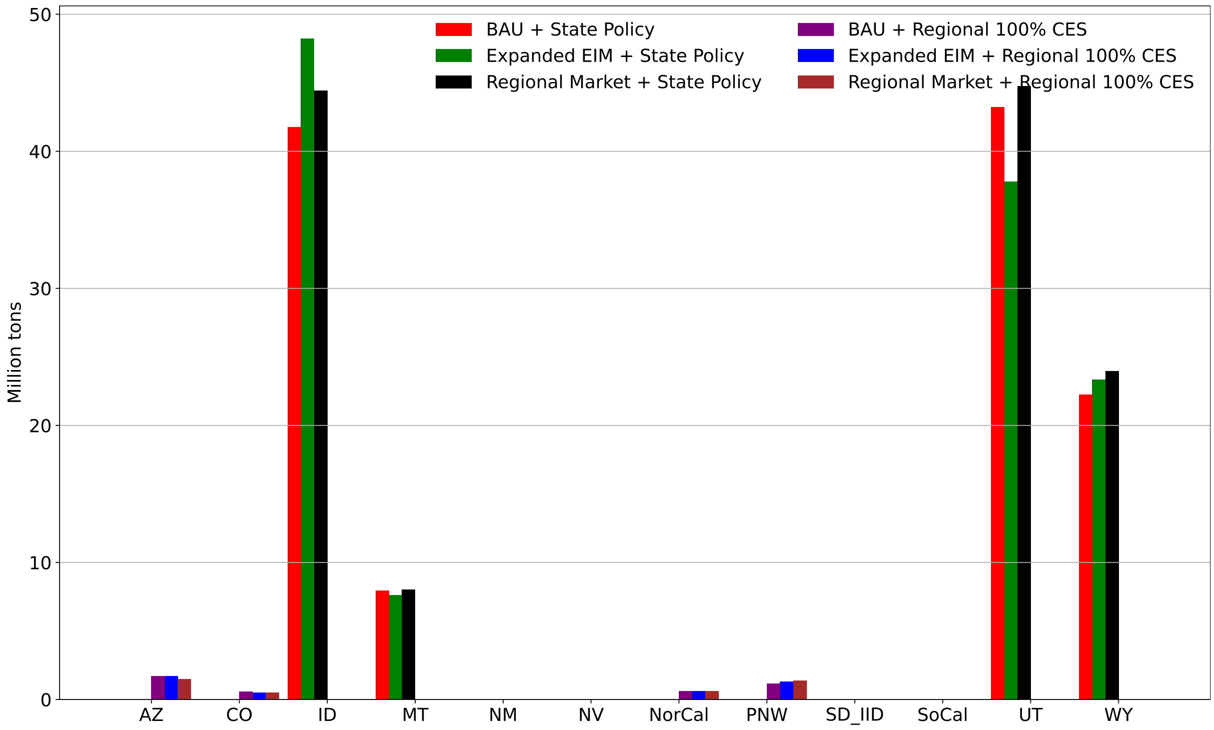

Supplemental Figure 19: GHG emission for all scenarios

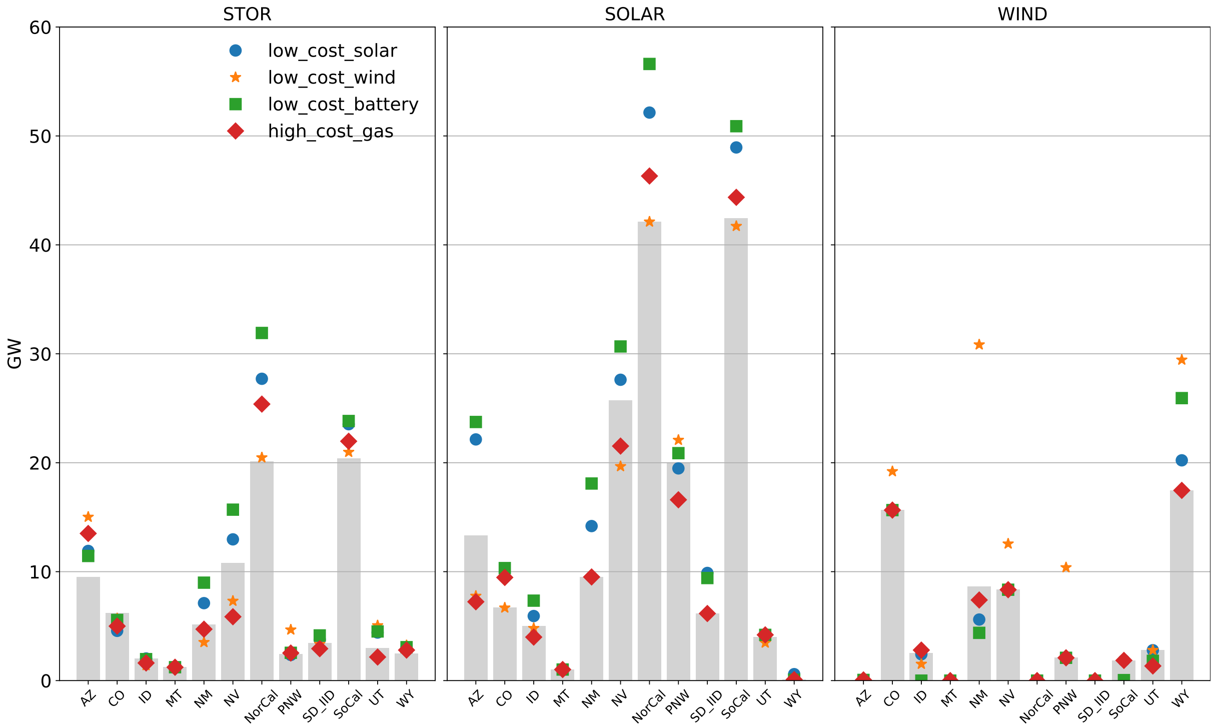

Supplemental Figure 20: Capacity change with respect to reference cost case for 2050 for BAU + Regional 100% CES scenario. Gray bar represents capacity for the reference cost case. Storage, solar, and wind capacity changes are shown in left, middle, and right panels, respectively.

Zones	Transmission Coordination		Limited Transmission Coordination	
	Existing + Expanded		Existing + Expanded (in Proposed Projects Paths)	
	BAU	Regional Market	BAU	Regional Market
Arizona	13,310	7,140	13,403	7,314
Colorado	6,701	6,683	8,825	9,082
Idaho	5,025	6,435	5,828	6,116
Montana	1,017	1,017	1,017	1,017
New Mexico	9,514	14,186	9,514	10,608
Nevada	25,716	25,716	25,406	25,915
North California	42,118	42,118	41,891	40,447
Pacific Northwest	19,971	20,616	18,592	19,928
San Diego	6,158	6,309	6,158	6,158
South California	42,442	43,915	43,766	43,951
Utah	4,006	1,240	2,915	1,199
Wyoming	92	92	92	92
**Sum**	**176,070**	**175,467**	**177,407**	**171,827**

Supplemental Table 42: Solar capacities (MW) with proposed projects and market configurations (Regional 100% CES)

Zones	Transmission Coordination		Limited Transmission Coordination	
	Existing + Expanded		Existing + Expanded (in Proposed Projects Paths)	
	BAU	Regional Market	BAU	Regional Market
Arizona	9,511	1,051	10,063	938
Colorado	6,216	3,367	5,734	5,513
Idaho	2,043	1,735	1,989	1,536
Montana	1,252	312	1,265	1,578
New Mexico	5,141	7,393	4,793	5,421
Nevada	10,783	11,517	10,260	10,684
North California	20,133	21,441	19,857	19,915
Pacific Northwest	2,451	2,687	2,887	1,661
San Diego	3,444	3,557	3,263	3,383
South California	20,375	21,460	21,529	22,884
Utah	3,002	226	4,455	289
Wyoming	2,498	1,818	2,246	1,369
**Sum**	**86,848**	**76,564**	**88,340**	**75,170**

Supplemental Table 43: Storage capacities (MW) with proposed projects and market configurations (Regional 100% CES)

Zones	Transmission Coordination		Limited Transmission Coordination	
	Existing + Expanded		Existing + Expanded (in Proposed Projects Paths)	
	**BAU**	**Regional Market**	**BAU**	**Regional Market**
Arizona	63	63	63	63
Colorado	15,653	15,653	15,653	15,653
Idaho	2,528	1,395	1,906	1,064
Montana	0	0	0	0
New Mexico	8,619	11,553	7,062	10,117
Nevada	8,344	8,344	8,344	8,344
North California	0	0	0	0
Pacific Northwest	2,093	2,093	2,093	2,093
San Diego	0	0	0	0
South California	1,853	1,853	1,853	1,853
Utag	2,789	2,789	2,789	2,789
Wyoming	17,462	19,232	17,462	17,462
**Sum**	**59,403**	**62,974**	**57,224**	**59,437**

Supplemental Table 44: Wind capacities (MW) with proposed projects and market configurations (Regional 100% CES)

Paths	Transmission Coordination		Limited Transmission Coordination	
	Existing + Expanded		Existing + Expanded (in Proposed Projects Paths)	
	**BAU**	**Regional Market**	**BAU**	**Regional Market**
AZ_to_NM	354	3,259	226	2,716
AZ_to_NV	0	0	0	0
AZ_to_SD_IID	0	0	0	0
AZ_to_SoCal	0	0	0	0
AZ_to_UT	0	0	0	0
CO_to_NM	1,455	1,893	0	0
CO_to_UT	700	348	0	0
CO_to_WY	1,128	1,287	850	850
ID_to_MT	0	0	0	0
ID_to_NV	0	0	0	310
ID_to_PNW	0	0	1,173	960
ID_to_UT	0	0	0	0
ID_to_WY	609	1,705	3,000	3,000
MT_to_PNW	0	0	0	0
MT_to_WY	3,687	3,654	0	0
NM_to_UT	0	0	0	0
NV_to_NorCal	0	0	0	0
NV_to_PNW	0	0	0	0
NV_to_SoCal	0	0	0	0
NV_to_UT	0	0	0	0
NorCal_to_PNW	0	0	0	0
NorCal_to_SoCal	0	0	0	0
PNW_to_SoCal	0	0	0	0
SD_IID_to_SoCal	0	0	0	0
SoCal_to_UT	0	0	0	0
UT_to_WY	0	0	1,208	941
NV_to_WY	NA	NA	225	511
**Sum**	**7,934**	**12,147**	**6,682**	**9,287**
Existing Grid (MW)	43,623	43,623	43,623	43,623
Total Cost ($B/year)	41.98	41.61	42.09	41.73

Supplemental Table 45: Expansion in transmission paths (MW) with proposed projects and market configurations (Regional 100% CES). NV_to_WY is one of the proposed projects' path picked by the model for expansion

References

[1] EIA: Form EIA-860 detailed data with previous form data (EIA-860A/860B) (2022). https://www.eia.gov/electricity/data/eia860/ Accessed 2022-07-08

[2] Palmintier, B.S., Webster, M.D.: Heterogeneous Unit Clustering for Efficient Operational Flexibility Modeling. IEEE Transactions on Power Systems **29**(3), 1089–1098 (2014). https://doi.org/10.1109/TPWRS.2013.2293127

[3] Jenkins, J.D., Sepulveda, N.A.: Enhanced Decision Support for a Changing Electricity Landscape: The GenX Configurable Electricity Resource Capacity Expansion Model (2017). https://energy.mit.edu/wp-content/uploads/2017/10/Enhanced-Decision-Support-for-a-Changing-Electricity-Landscape.pdf

[4] Bezanson, J., Edelman, A., Karpinski, S., Shah, V.B.: Julia: A Fresh Approach to Numerical Computing. SIAM Review **59**(1), 65–98 (2017). https://doi.org/10.1137/141000671

[5] Dunning, I., Huchette, J., Lubin, M.: JuMP: A Modeling Language for Mathematical Optimization. SIAM Review **59**(2), 295–320 (2017). https://doi.org/10.1137/15M1020575

[6] Gurobi Optimization, L.: Gurobi Optimizer Reference Manual (2022). https://www.gurobi.com/ Accessed 2022-07-21

[7] SDSC: Triton Shared Computing Cluster. University of California San Diego (2022). https://www.sdsc.edu/index.html

[8] Schivley, G., Welty, E., Xu, Q., Patankar, N., Chakrabarti, S., Jacobson: PowerGenome. PowerGenome (2022). https://github.com/PowerGenome/PowerGenome Accessed 2022-05-19

[9] EPA: EPA's Power Sector Modeling Platform v6 using IPM Summer 2021 Reference Case (2018). https://www.epa.gov/power-sector-modeling/epas-power-sector-modeling-platform-v6-using-ipm-summer-2021-reference-case Accessed 2022-11-22

[10] Long, J.C., Baik, E., Jenkins, J.D., Kolster, C., Chawla, K., Olson, A., Cohen, A., Colvin, M., Benson, S., Jackson, R., et al.: Clean firm power is the key to california's carbon-free energy future. Issues in Science and Technology (2021)

[11] CEC: Input & Assumptions - CEC SB 100 Joint Agency Report (2020). https://efiling.energy.ca.gov/getdocument.aspx?tn=234525

[12] Ming, Z., Olson, A., De Moor, G., Jiang, H., Schlag, N.: Long-Run Resource Adequacy under Deep Decarbonization Pathways for California. Technical report, Energy and Environmental Economics, Inc. (June 2019). https://www.ethree.com/wp-content/uploads/2019/06/E3_Long_Run_Resource_Adequacy_CA_Deep-Decarbonization_Final.pdf

[13] NREL: Electricity Annual Technology Baseline (ATB) Data Download (2021). https://atb.nrel.gov/electricity/2021/data Accessed 2022-06-28

[14] Feldman, D., Margolis, R.: H2 2020 Solar Industry Update. Technical Report NREL/PR-7A40-79758, National Renewable Energy Laboratory (April 2021). https://doi.org/10.2172/1808491

[15] Zhuo, Z., Du, E., Zhang, N., Nielsen, C.P., Lu, X., Xiao, J., Wu, J., Kang, C.: Cost increase in the electricity supply to achieve carbon neutrality in China. Nature Communications **13**(1), 3172 (2022). https://doi.org/10.1038/s41467-022-30747-0. Number: 1 Publisher: Nature Publishing Group. Accessed 2022-11-14

[16] Wikipedia: List of energy storage power plants. Page Version ID: 1096409691 (2022). https://en.wikipedia.org/wiki/List_of_energy_storage_power_plants Accessed 2022-07-08

[17] Wikipedia: List of pumped-storage hydroelectric power stations. Page Version ID: 1096680113 (2022). https://en.wikipedia.org/wiki/List_of_pumped-storage_hydroelectric_power_stations Accessed 2022-07-08

[18] CEC: 2021 SB 100 Joint Agency Report. Technical report, California Energy Commission (March 2021). https://efiling.energy.ca.gov/EFiling/GetFile.aspx?tn=237167&DocumentContentId=70349

[19] Brownlee, B., Simonson, G., Fraser, K., Ramirez, D., Liotiris, C., Moyer, K.: Western Flexibility Assessment Investigating the West's Changing Resource Mix and Implications for System Flexibility. Technical report, Energy Strategies (December 2019). https://westernenergyboard.org/wp-content/uploads/2019/12/12-10-19-ES-WIEB-Western-Flexibility-Assessment-Final-Report.pdf

[20] Hadjerioua, B., Wei, Y., Kao, S.-C.: An Assessment of Energy Potential at Non-Powered Dams in the United States. Technical report, Oak Ridge National Laboratory (April 2012). https://www.ornl.gov/publication/assessment-energy-potential-non-powered-dams-united-states-0

[21] Hadjerioua, B., Wei, Y., Kao, S.-C.: US Hydropower Potential from Existing Non-powered Dams (greater than 1MW). Oak Ridge National Laboratory (ORNL), Oak Ridge, TN (United States) (2012). https://www.osti.gov/servlets/purl/1493305/ Accessed 2022-07-13

[22] Lopez, A., Roberts, B., Heimiller, D., Blair, N., Porro, G.: U.S. Renewable Energy Technical Potentials: A GIS-Based Analysis. Technical Report NREL/TP-6A20-51946,

National Renewable Energy Laboratory (July 2012). https://www.nrel.gov/docs/fy12osti/51946.pdf

[23] Stanley, A.: Mapping the U.S.-Canada Energy Relationship. Technical report, Center for Strategic and International Studies (May 2018). https://www.csis.org/analysis/mapping-us-canada-energy-relationship

[24] EIA: Mexico Week: U.S.-Mexico electricity trade is small, with tight regional focus (2013). https://www.eia.gov/todayinenergy/detail.php?id=11311 Accessed 2022-07-13

[25] WECC: Transfers by State (2019). https://www.wecc.org/epubs/StateOfTheInterconnection/Pages/Transfers-by-State.aspx Accessed 2022-06-30

[26] WECC: Total Disposition by State (2019). https://www.wecc.org/epubs/StateOfTheInterconnection/Pages/Total-Disposition-by-State.aspx Accessed 2022-06-30

[27] NERC: 2022 Summer Reliability Assessment. Technical report, North American Electric Reliability Corporation (May 2022). https://www.nerc.com/pa/RAPA/ra/ReliabilityAssessmentsDL/NERC_SRA_2022.pdf Accessed 2022-12-15

[28] Johnston, J., Henriquez-Auba, R., Maluenda, B., Fripp, M.: Switch 2.0: A modern platform for planning high-renewable power systems. SoftwareX **10**, 100251 (2019). https://doi.org/10.1016/j.softx.2019.100251

[29] NREL: Western Wind and Solar Integration Study (2017). https://www.nrel.gov/grid/wwsis.html Accessed 2022-07-13

[30] Carr, T., Dykema, A., Brian, T., Worley, C., Little, D., Blend, J., Usibelli, T., Moyer, K., Liotiris, C., Fraser, K., Kimball, B.: Western Outreach

Project Report. Technical report, Energy Strategies, LLC (October 2016). https://static1.squarespace.com/static/59b97b188fd4d2645224448b/t/59f40357652dea26877e092e/1509163867513/RETI+2+Western+Outreach+Project+Report.pdf

[31] Gorman, W., Mills, A., Wiser, R.: Improving estimates of transmission capital costs for utility-scale wind and solar projects to inform renewable energy policy. Energy Policy **135**, 110994 (2019). https://doi.org/10.1016/j.enpol.2019.110994. Accessed 2022-06-06

[32] CAISO: 20-Year Transmission Outlook. Technical report, California ISO (January 2022). http://www.caiso.com/InitiativeDocuments/Draft20-YearTransmissionOutlook.pdf

[33] BPA: Montana Renewables Development Action Plan (2018). https://www.bpa.gov/-/media/Aep/projects/montana-renewable-resource-action-plan/montana-renewables-development-action-plan-june-2018.pdf

[34] Perez, A.P., Sauma, E.E., Munoz, F.D., Hobbs, B.F.: The Economic Effects of Interregional Trading of RenewableEnergy Certificates in the U.S. WECC. The Energy Journal **37**(4) (2016). https://doi.org/10.5547/01956574.37.4.aper

[35] Sergi, B., Cole, W.: Operating reserves in reeds. Technical report, National Renewable Energy Lab.(NREL), Golden, CO (United States) (2021). https://www.nrel.gov/docs/fy22osti/81706.pdf

[36] Lew, D., Brinkman, G., Ibanez, E., Florita, A., Heaney, M., Hodge, B.-M., Hummon, M., Stark, G., King, J., Lefton, S.A., et al.: Western wind and solar integration study phase 2. Technical report, National Renewable Energy Lab.(NREL), Golden, CO (United States) (2013). https://www.nrel.gov/docs/fy13osti/55588.pdf

[37] CPUC: System Reliability Modeling Datasets 2023 (2023). https://www.cpuc.ca.gov/industries-and-topics/electrical-energy/electric-power-procurement/long-term-procurement-planning/2022-irp-cycle-events-and-materials/system-reliability-modeling-datasets-2023 Accessed 2024-10-10

[38] CESA: Table of 100% Clean Energy States (2024). https://www.cesa.org/projects/100-clean-energy-collaborative/guide/table-of-100-clean-energy-states/ Accessed 2024-10-22

[39] NCEL: Colorado Sets Targets to Eliminate Greenhouse Gas Emissions by 2050 (2023). https://www.ncelenviro.org/articles/colorado-sets-targets-to-eliminate-greenhouse-gas-emissions-by-2050/ Accessed 2024-10-22

[40] NEC: Taking Action to Ensure the Federal Government Does Its Part to Help California Meet Its Electricity Needs (2020). https://clintonwhitehouse5.archives.gov/WH/EOP/nec/html/doc080300.html Accessed 2024-10-22
